# Supplementary material for: Identifying a key spot for electron mediator-interaction to tailor CO dehydrogenase’s affinity
Source: Nat Commun. 2024 Mar 28;15:2732. doi: 10.1038/s41467-024-46909-1 (PMC10979024; doi:10.1038/s41467-024-46909-1)
Supplement: Supplementary file 1 — Supplementary information [file 41467_2024_46909_MOESM1_ESM.pdf]

Supplementary Information

Identifying a key spot for electron mediator-interaction to tailor CO dehydrogenase's affinity

Suk Min Kim[1†*], Sung Heuck Kang[1†], Jinhee Lee[1†], Yoonyoung Heo[2†], Eleni G. Poloniataki[1], Jingu Kang[1], Hye-Jin Yoon[2], So Yeon Kong[2], Yaejin Yun[2], Hyunwoo Kim[1], Jungki Ryu[1], Hyung Ho Lee[2*] and Yong Hwan Kim[1,3*]

[1]School of Energy and Chemical Engineering, Ulsan National Institute of Science and Technology (UNIST), 50 UNIST-gil, Ulsan 44919, Republic of Korea

[2]Department of Chemistry, College of Natural Sciences, Seoul National University, 1 Gwanak-ro, Gwanak-gu, Seoul 08826, Republic of Korea

[3]Graduate School of Carbon Neutrality, Ulsan National Institute of Science and Technology (UNIST), 50 UNIST-gil, Ulsan 44919, Republic of Korea

[†]These authors contributed equally.

[*]These authors jointly supervised this work.

Supplementary Tables 1–10

Supplementary Table 1 | CO and CO_2 utilizing enzymes

Group	Name (EC number)	Classification	Origin	PDB code	Mediator use	Reference
Carbon monoxide dehydrogenase (CODH)	Ni–Fe CODH (1.2.7.4)	Homodimeric CODH	*Carboxydothermus hydrogenoformans*	Oxygen-sensitive type: 1SU6, 1SU7, 1SU8, 1SUF, 3B51, 3B52, 3B53, 3I39, 5FLE, 2YIV, 4UDX, 4UDY Less oxygen-sensitive type: 6ELQ, 7ERR, 7XDM, 7XDN, 7XDP	BV, EV	This study
			Desulfovibrio vulgaris	6B6V, 6B6W, 6B6X, 6B6Y, 6DC2, 6ONC, 6OND, 6ONS, 6VWY, 6VWZ, 6VX0, 6VX1	MV	Source[1]
			Rhodospirillum rubrum	1JQK	BV, MV	Source[2]
			Thermococcus onnurineus NA1	–	BV, EV	This study
			Thermococcus sp. AM4	6T7J	MV	Source[3]
		Heteromeric ACS/CODH complex	*Carboxydothermus hydrogenoformans*	7ZKJ, 7ZKK, 7ZKV	MV	Source[4]
			Clostridium autoethanogenum	6YTT, 6YU9, 6YUA	MV	Source[5]
			Methanosarcina barkeri	3CF4	MV	Source[6]
			Moorella thermoacetica	3I01, 3I04, 1MJG, 1OAO, 6X5K, 2Z8Y	MV	Source[7]
Formate dehydrogenase (FDH)	Non-metal FDH (1.17.1.9)	Non-metal containing	*Arabidopsis thaliana*	3JTM, 3N7U, 3NAQ	NAD^+	Source[8]
			Candida boidinii	2FSS, 2J6I, 5DN9, 5DNA, 6D4B, 6D4C	NAD^+	Source[9]
			Chaetomium thermophilum	6T8Y, 6T8Z, 6T92, 6T94	NAD^+	Source[10]
			Granulicella mallensis	4XYB, 4XYG, 6T8C, 6T8J, 6T9W, 6T9X, 6TB6	$NADP^+$	Source[11]
			Moraxella sp.C-1	2GSD, 3FN4	NAD^+	Source[12]
			Physcomitrium patens	7ARZ		
			Pseudomonas sp. 101	2GO1, 2GUG, 2NAC, 2NAD, 6JUJ, 6JUK, 6JWG, 6JX1	NAD^+, NAD	Source[13]
			Thiobacillus sp. KNK65MA	3WR5	NAD^+	This study
	Mo FDH (1.17.1.9)	Mo (molybdenum) containing	*Cupriavidus necator*	6VW7, 6VW8	BV, EV, NAD^+	Source[14] Source[15]
			Escherichia coli	1AA6, 1FDI, 1FDO, 1KQF, 1KQG, 2IV2	BV	Source[16]
			Methanospirillum hungatei	7BKB, 7BKC, 7BKC, 7BKD, 7BKE	BV	Source[17]
			Rhodobacter capsulatus	6TG9, 6TGA	EV	This study
	W FDH (1.17.1.9)	W (tungsten) containing	*Desulfovibrio gigas*	1H0H	BV, MV	Source[18]
			Desulfovibrio vulgaris	6SDR, 6SDV	BV, MV	Source[19,20]
			Methanothermobacter wolfeii	5T5I, 5T5M, 5T61		
			Methylobacterium extorquens	8J83, 7XQW	BV, EV, NAD^+	This study

Supplementary Table 2 | Numbers of surface aromatic residues in viologen-based CODHs

Group	Metal at D-cluster	Organism	Name (PDB code)	No. of surface exposed F/Y/W	No. of residues within 8 Å of Fe-S clusters	No. of putative site
Ni–Fe CODHs	4Fe4S	*Carboxydothermus hydrogenoformans*	*Ch*CODH2 (1SU7)[21]	10	3	2
		Carboxydothermus hydrogenoformans	*Ch*CODH4 (6ELQ)[22]	22	3	1
		Rhodospirillum rubrum	*Rr*CODH (1JQK)[23]	17	5	2
		Moorella thermoacetica	*Mt*CODH (1MJG)[24]	22	3	1
	2Fe2S	*Desulfovibrio vulgaris*	*Dv*CODH (6OND)[25]	14	5	2

Supplementary Table 3 | Summary of synthesized, cloned DNAs and the used primers in this study

a. Synthesised DNAs

CODH (vector)	DNA sequence	Reference
*Ch*CODH2 (pET28a)	ATGGCGAAACAAAATCTGAAGAGCACCGACCGTGCGGTTCAACAAATGCTGGATAAAGCGAAGCGTGAGGGTATTCA<br>AACCGTGTGGGATCGTTACGAGGCGATGAAGCCGCAGTGCGGTTTCGGCGAAACCGGTCTGTGCTGCCGTCACTGCC<br>TGCAAGGTCCGTGCCGTATTAACCCGTTTGGCGATGAGCCGAAAGTGGGCATTTGCGGTGCGACCGCGGAAGTGATC<br>GTTGCGCGTGGTCTGGACCGTAGCATTGCGGCGGGTGCGGCGGGTCATAGCGGTCATGCGAAGCACCTGGCGCACAC<br>CCTGAAGAAAGCGGTGCAGGGCAAAGCGGCGAGCTATATGATTAAGGACCGTACCAAACTGCACAGCATCGCGAAGC<br>GTCTGGGTATTCCGACCGAAGGCCAAAAAGACGAGGATATTGCGCTGGAAGTTGCGAAAGCGGCGCTGGCGGACTTC<br>CATGAGAAAGATACCCCGGTTCTGTGGGTGACCACCGTTCTGCCGCCGAGCCGTGTGAAGGTTCTGAGCGCGCATGG<br>TCTGATCCCGGCGGGTATTGATCACGAAATCGCGGAGATTATGCACCGTACCAGCATGGGTTGCGACGCGGATGCGC<br>AGAACCTGCTGCTGGGTGGCCTGCGTTGCAGCCTGGCGGACCTGGCGGGTTGCTACATGGGCACCGACCTGGCGGAT<br>ATCCTGTTTGGTACCCCGGCGCCGGTGGTTACCGAAAGCAACCTGGGCGTGCTGAAGGCGGATGCGGTGAACGTTGC<br>GGTGCACGGTCACAACCCGGTTCTGAGCGACATCATTGTTAGCGTGAGCAAAGAGATGGAAAACGAGGCGCGTGCGG<br>CGGGTGCGACCGGTATCAACGTGGTTGGTATTTGCTGCACCGGCAACGAGGTGCTGATGCGTCACGGTATTCCGGCG<br>TGCACCCACAGCGTTAGCCAGGAAATGGCGATGATCACCGGCGCGCTGGACGCGATGATCCTGGATTATCAGTGCAT<br>TCAACCGAGCGTGGCGACCATTGCGGAGTGCACCGGTACCACCGTTATTACCACCATGGAAATGAGCAAAATCACCG<br>GTGCGACCCATGTGAACTTTGCGGAGGAAGCGGCGGTTGAGAACGCGAAGCAAATCCTGCGTCTGGCGATTGATACC<br>TTTAAACGTCGTAAGGGTAAACCGGTGGAGATCCCGAACATTAAGACCAAAGTGGTTGCGGGCTTCAGCACCGAAGC<br>GATCATTAACGCGCTGAGCAAGCTGAACGCGAACGATCCGCTGAAACCGCTGATTGACAACGTGGTTAACGGTAACA<br>TCCGTGGCGTGTGCCTGTTCGCGGGTTGCAACAACGTTAAGGTGCCGCAGGACCAAAACTTTACCACCATTGCGCGT<br>AAGCTGCTGAAACAGAACGTTCTGGTGGTTGCGACCGGTTGCGGTGCGGGTGCGCTGATGCGTCACGGTTTTATGGA<br>CCCGGCGAACGTGGATGAGCTGTGCGGCGACGGTCTGAAAGCGGTTCTGACCGCGATCGGTGAAGCGAACGGTCTGG<br>GTGGCCCGCTGCCGCCGGTGCTGCACATGGGTAGCTGCGTTGACAACAGCCGTGCGGTGGCGCTGGTTGCGGCGCTG<br>GCGAACCGTCTGGGCGTTGACCTGGATCGTCTGCCGGTGGTTGCGAGCGCGGCGGAAGCGATGCATGAGAAGGCGGT<br>GGCGATTGGTACCTGGGCGGTTACCATCGGTCTGCCGACCCACATTGGTGTGCTGCCGCCGATCACCGGCAGCCTGC<br>CGGTGACCCAAATCCTGACCAGCAGCGTTAAAGATATTACCGGTGGCTACTTCATCGTTGAACTGGACCCGGAGACC<br>GCGGCGGACAAACTGCTGGCGGCGATCAATGAGCGTCGTGCGGGTCTGGGTCTGCCGTGGTGA	Source[26]
*Ch*CODH4 (pET28a)	ATGGATAAAAGCAAGTTATCAGTAGATCCTGTGATCCCGAATTTATATCGTAAAGCCAGAGAGGAAGGGATTTCCAC<br>TGTTTTTGATCGTTATGAAGCACAGCAGCCTCAGTGCGGATTTGGGCTTACAGGCCTTTGCTGTCGTCATTGCGTGC<br>AGGGACCGTGCCGCATAGATCCGTTTGGGGAAGGACCGCAAGCGGGCATTTGCGGTGCTACCGCCGAGGTAATAACG<br>GCTCGCAATCTGCTGCGTCAAGTCACGGCGGGTGCTGCCGCCCATGTAGACCATGCGTATGATGTGCTGGAAGTTTT<br>GGAACAAATCGCCCAAGGTACGGAATCATACAGTATCAAAGATCAAGAAAAGTTAAAGCAGGTCGCTTTTACCTTAG<br>GTATAGATACCGCTAACAAAACAGAGCAGGAGATTGTTGAAGAGATGTGCCAAATTATCTATCGGGATTTTGCCAAT<br>TCTGGTGCAACACCGATGACCTATCTGAAAGCCAACTCTCCTCGGGAACGCCTTGAGACATGGGAAAAATTAGGGGT<br>TCTGCCTCGTAACCCAGACCGTGAAATCAGAGAAGCCTTACACCAGACTACAATGGGGATGGATGCGGACCCAGTAA<br>ATTTAATCTTAAAAACTATTCGCCTTGGTCTGGTTGATGGTTTTGCGGGTCTCAAGTTAGCGACGGATTTACAGGAC<br>ATCATTTTTGGCACTCCCCAGCCAGTTGTCACAGAAGCAAATCTGGGTGTACTCAAAGAAGATTATGTGAATATCAT<br>AGTCCACGGCCATGTTCCGTTACTTAGCGAAAAAATTGTTGAATGGAGCAGAAAGTTGGAAGATGAGGCGAAAAAAG<br>CAGGGGCAAAAGGAATCAATCTTGCAGGTATATGTTGTACTGGTAACGAGGTGTTAATGCGTCAGGGTGTACCGTTA<br>GCAACAAACTTCCTGGCCCAGGAGTTGGCGATTATTACGGGGGCAGTTGACTTAATGGTAGTGGATGTTCAGTGTAT<br>TATGCCCTCATTAGCAGAAATTGCTGCTTGCTATCATACCCGCTTAGTCACTACCATGCCGATTGTGAAAATTCCAG<br>GTGCGGAGCATGTTCCGTTCACCACAGAAACTGCCGATGAGGCCTCGCAGCAGATTGTGCGTATGGCTATAGAGAGT<br>TACCAAAAACGCAATCCAGCCAAGGTCTACATCCCCAGGGAAAAAGCCAAAGTTGTAGCAGGATTCAGCGTTGAGGC<br>AATAGTAAAAGCGTTAGCGAAACTAAATCCTGACGATCCGTTAAAGCCCTTAATTGATAATATTGTGTCTGGCAACA<br>TTCTGGGTGTGGTCGCCACCGTTGGTTGTAACAATGTGAAGGTCAAGCATGATTGGTTCCACATAGAATTAGTAAAA<br>GAACTAATTAAAAACAATGTGTTAGTGGTGACTACAGGCTGTTCCGCTCATGCGTTAGCGAAAGCAGGCTTGATGGA<br>CCCCGCAGCAGCCGAATGGGCTGGCGAGGGTTTACGAGCCGTCTTAACGGCAATAGGCACGGCTAATGATTTGGGCG<br>GCCCGCTGCCGCCTGTTTTGCACATGGGATCGTGTGTTGATAACTCCCGAATCGGGGACTTAGTGATCGCCGTCGCA<br>AACTACCTTAAAGTAAGTCCAAAAGACCTTCCGATTGCGGCTTCTGCACCTGAATACCAGCATGAAAAGGCTTTGAG<br>CATCGGAACCTGGGCGGTAGCGATGGGTATTATGACCCACTTGGGAGTTGTTCCACCTGTGGTCGGAAGTTCAAAAG<br>TTACCCGTATTCTTACCCAAGATGCCGAGGCTTTAATAGGCGGCAAATTTTATGTGGAAACGGACCCATATAAAGCA<br>GCAGCGGGCATCATTGAACATATTAAGGCTAAACGGGCTCTACTGAATTTATAA	Source[26]
*Dv*CODH (pET28a)	ATGAGTTCTAGTAAGACTATCCGTAGCCGTTCGATTTGGGATGATGCACATGCAATGCTCGAAAAAGCGAAAGCAGA<br>GGGTATCTCCACCGTCTGGGATCGAGCTGCGGAACAGACGCCAGCCTGTAAATTCTGTGAATTGGGCACCACCTGCC<br>GCAATTGCATCATGGGCCCGTGCCGTATCGCCAACCGCAAGGACGGAAAGATGAGACTTGGCGTTTGTGGAGCAGAT<br>GCCGATGTAATCGTGGCGCGAAATTTTGGCCGTTTCATCGCTGGAGGAGCAGCTGGTCACTCAGATCACGGACGCGA<br>CCTGATCGAAACATTGGAAGCCGTAGCCGAAGGCAAAGCTCCTGGCTATACGATTCGTGACGTCGCAAAATTGAGAA<br>GAATCGCGGCCGAATTGGGCGTAGCCGACGCAGCGACACGCCCCGCTCATGACGTGGCCGCTGACCTCGTCACCATA<br>TGTTATAACGACTTCGGCAGCCGGCGTAATGCTCTGGCCTTTCTGGCGCGTGCACCGCAGGTGCGGCGCGACTTGTG<br>GCAACGCCTTGGCATGACCCCACGTGGCGTAGACCGCGAGATTGCCGAAATGATGCACCGCACTCATATGGGCTGTG<br>ATAACGACCACACATCTCTGCTCGTACATGCTGCGCGGACGGCGCTCGCAGACGGTTGGGGAGGTTCTATGATAGGC<br>ACTGAATTGTCCGATATTCTATTTGGCACGCCTAGACCGAGACAGTCAACAGTCAATCTCGGTGTCTTGCGCAAAGA<br>TGCCGTCAACATCTTAGTTCATGGACATAACCCAGTCGTCTCCGAAATGATCCTGGCCGCCACACGTGAACCGGCTG<br>TAAGGCAGGCTGCACAGGACGCCGGCGCAGCAGACATCAATGTCGCGGGGCTATGTTGCACAGGTAACGAGCTGTTG<br>ATGCGACAGGGTATTCCCATGGCAGGCAACCACCTCATGACCGAACTCGCTATTGTCACAGGTGCGGCCGATGCAAT<br>TGTCGCAGATTATCAATGTATAATGCCAAGCCTGGTGCAGATTGCTGCGTGTTACCACACCCGCTTTGTAACGACTT<br>CTCCTAAAGGGCGTTTTACTGGGGCCACTCATGTGGAGGTGCATCCGCACAATGCGCAAGAGAGGTGCCGTGAGATC<br>GTGATGCTGGCAATTGATGCGTACACCAGACGAGATCCCGCGCGGGTTGACATACCGTCGCAACCCGTGTCAATAAT<br>GTCCGGGTTTTCCAATGAGGCCATTTTAGAAGCCCTTGGCGGCACTCCAAAACCTCTCATCGATGCTGTTGTGGCAG<br>GGCAAATACGGGGATTTGTGGGCATCGTGGGTTGCAATAATCCAAAAATTCGTCAGGATTCAGCTAATGTAACGCTC<br>ACGCGGGAACTGATACGCCGCGACATAATGGTGCTTGCCACAGGATGTGTCACGACGGCTGCTGGCAAGGCCGGACT<br>GCTGGTCCCTGAAGCTGCATCGAAAGCAGGCGAGGGGCTGGCCGCCGTGTGCCGCAGTCTTGGCGTGCCTCCGGTGC<br>TGCATATGGGCAGCTGCGTTGATAATTCTCGCATCTTGCAGTTGTGCGCCCTGCTGGCAACCACTCTGGGCGTTGAC<br>ATATCCGATCTGCCCGTGGGGGCCAGTTCACCAGAATGGTATTCCGAGAAAGCAGCGGCAATTGCCATGTATGCCGT<br>GGCAAGCGGCATTCCCACGCATCTTGGTCTTCCGCCCAATATCTTAGGCAGCGAGAACGTAACCGCCATGGCCCTGC<br>ATGGGCTACAGGACGTGGTAGGAGCGGCCTTCATGGTTGAACCGGATCCTGTCAAGGCGGCTGATATGCTGGAAGCG<br>CATATCGTGGCACGCCGCGCAAGGCTTGGTCTCACATCATAA	Source[26]

*Rr*CODH<br>(pET28a)	ATGACGCATCATGACTGTGCCCATTGTTCCTCCGATGCCTGCGCGACCGAAATGTTGAATTTAGCCGAGGCCAACAG<br>CATAGAAACCGCTTGGCATCGCTACGAAAAGCAGCAGCCCCAATGCGGCTTTGGCTCGGCCGGCCTGTGTTGCCGCA<br>TCTGTTTAAAAGGCCCATGTCGCATCGATCCCTTTGGCGAAGGCCCGAAGTACGGAGTCTGTGGCGCCGACCGCGAC<br>ACCATCGTCGCCAGGCATCTGGTGCGCATGATTGCGGCCGGCACCGCAGCCCATTCCGAGCACGGCCGGCACATTGC<br>CCTGGCCATGCAACATATTTCACAAGGCGAGTTACACGATTATAGCATCCGCGATGAGGCCAAGCTCTATGCCATCG<br>CCAAGACACTGGGCGTGGCCACAGAGGGACGTGGTCTGTTAGCGATCGTAGGCGATTTGGCGGCGATAACTCTGGGA<br>GACTTCCAGAATCAGGATTACGATAAACCTTGCGCTTGGCTTGCAGCGTCTCTCACGCCGCGTCGGGTGAAGCGCTT<br>GGGTGATCTGGGTCTGCTGCCACACAATATTGATGCTAGCGTTGCGCAGACGATGTCACGCACTCATGTGGGTTGCG<br>ATGCCGATCCAACCAATCTGATTCTGGGTGGCTTGAGGGTTGCCATGGCCGATCTTGACGGTTCAATGCTGGCGACA<br>GAGTTATCTGATGCACTGTTTGGCACGCCACAGCCTGTGGTTAGTGCCGCCAATCTGGGTGTCATGAAGCGCGGAGC<br>CGTAAATATAGCCGTCAATGGTCATAACCCGATGCTCAGCGATATCATTTGTGATGTGGCAGCGGATCTGCGAGACG<br>AAGCAATCGCCGCTGGGGCAGCCGAGGGGATTAACATCATTGGCATTTGCTGTACCGGCCATGAAGTTATGATGCGC<br>CATGGCGTTCCGCTTGCCACCAATTACTTATCGCAGGAATTACCTATCCTGACCGGCGCATTAGAGGCGATGGTCGT<br>CGATGTACAGTGTATAATGCCGAGTCTTCCTCGCATTGCCGAATGCTTCCACACCCAGATCATCACCACCGACAAAC<br>ACAATAAAATCAGTGGCGCTACCCATGTGCCCTTCGATGAGCACAAGGCAGTTGAGACTGCCAAGACGATTATTAGA<br>ATGGCGATTGCCGCCTTTGGACGGCGTGATCCTAACCGCGTCGCTATTCCTGCTTTTAAACAGAAATCAATTGTAGG<br>CTTTTCAGCCGAAGCAGTCGTCGCCGCCCTGGCCAAAGTTAATGCGGACGATCCGCTAAAGCCGCTGGTTGATAATG<br>TTGTAAACGGCAATATACAAGGTATAGTGCTTTTTGTCGGCTGCAACACGACCAAGGTTCAGCAAGACAGCGCGTAT<br>GTAGATCTAGCAAAGTCTCTCGCCAAGCGCAATGTGCTTGTGCTTGCAACTGGCTGTGCCGCCGGCGCCTTCGCCAA<br>AGCTGGATTAATGACCTCCGAAGCGACCACCCAATATGCTGGCGAAGGCTTGAAAGGGGTACTGTCAGCCATC GGTA<br>CGGCCGCCGGTCTGGGAGGGCCACTGCCATTGGTCATGCACATGGGCTCTTGCGTCGATAACAGTCGAGCGGTTGCT<br>TTGGCCACAGCTTTAGCCAATAAGTTAGGTGTAGATCTCTCTGATCTGCCGCTGGTCGCCAGTGCTCCGGAATGCAT<br>GAGCGAGAAGGCCCTGGCCATCGGCTCGTGGGCGGTGACTATTGGTCTGCCGACCCATGTCGGCTCGGTTCCTCCGG<br>TCATAGGTTCGCAGATCGTGACTAAGCTGGTGACCGAAACGGCAAAAGACTTGGTGGGCGGTTATTTTATCGTTGAT<br>ACCGACCCCAAAAGTGCCGGGGACAAGCTTTATGCCGCAATTCAAGAACGTAGAGCCGGGCTTGGCCTGTAA	This study
*Rr*CooCTJ<br>(pCDFDuet)	ATGAAAATTGCGGTGACTGGGAAAGGCGGTGTTGGCAAGTCCACTATCGTTGGTATGCTGGCACGTGCTTTGAGCGA<br>CGAGGGTTGGAGGGTGATGGCAATTGATGCCGATCCGGATGCGAATTTAGCTTCTGCCATTGGCGTACCGGCTGAAA<br>GACTAAGCGCGCTTTTACCGATATCAAAAATGACCGGTTTAGCCCGCGAACGGACCGGGGCTTCCGAAACAACGGGC<br>ACCCATTTTATTCTAAACCCTCGGGTTGATGATATTCCTGAACAGTTTTGTGTAGACCATGCAGGAATAAAACTGCT<br>GTTGATGGGAACGGTTAATCACGCCGGTTCTGGTTGCGTCTGTCCAGAACATGCCCTGGTACGCACACTGCTGCGAC<br>ATATACTGACTAAACGTAAGGAATGTGTTTTAATTGACATGGAAGCTGGAATCGAGCACTTCGGCCGTGGGACAATT<br>GAAGCGGTAGATTTGTTAGTGATAGTGATCGAGCCTGGCTCTAGAAGTTTACAGACAGCTGCTCAGATAGAAGGTCT<br>GGCACGCGATCTGGGGATCAAAACGATCTGTCATATTGCTAATAAACTGGCTTCGCCCGTCGATGTGGGCTTTATTC<br>TTGATCGTGCAGACCAATTCGATCTTTTGGGAAGTATTCCATTTGACAGCGCAATCCAAGCTGCAGACCAGGCCGGT<br>CTGTCATGCTATGATCTTTCACCGGCATGCCGTGATAAGGCACACGCCCTGATGGCCGCTTTACTTGAACGCGTC GG<br>TCCAACCCAGGGAGTTAGTTAAataaggagatataccATGTGTATGGCAAAAGTTGTGTTAACCAAAGCAGATGGTG<br>GACGCGTAGAAATCGGTGATGTTTTGGAAGTACGTGCCGAAGGCGGTGCGGTGCGGGTCACTACACTGTTTGATGAA<br>GAACATGCTTTTCCGGGGCTGGCTATTGGACGTGTTGACCTGAGATCAGGCGTTATTTCACTTATAGAGGAGCAGAA<br>TCGTTAAataaggagatataccATGACCGAATCTCCTGAACGTGGTCGTAAAAGATTAGGAATATATTTAGCGCATT<br>TTTTAGATCATGTTGAGGGTCACATGGGCGAGATTGGGGTTCAGCGTGATGCTTTGGCCGAAGATGCTCGGTTAGGT<br>GCATTAATCGATCGTGCATTAGCAGATATGGCTGTGGCTCGCGCCTCGCTTAATGCAGTCTTACGCGATTTAGATGG<br>AGAAGCACCGGCACCGGCAAGCCCCGAAGCAGTACATAGTCCATTTCATTCCCATGCACATTCTCACGACCACGATC<br>ATGCACACGGCCATTCACATGACCATGCCCACGATCATTGTCATTGCCATGATCATCCTTAA	This study

b. Cloned genes of CODH and FDHs

Name	Origin	Gene (locus tag)	Vector	Host*	Reference
*To*CODH	*Thermococcus onnurineus* NA1	*cooS* (TON_1018)	pET28a	*Escherichia coli* BL21	Source[27]
*Me*FDH1	*Methyloacterium extorquens* AM1	*fdh1AB* (MexAM1_META1p5031–5032)	pCM110	*M. extorquens* AM1	Source[28]
*Rc*FDH	*Rhodobacter capsulatus*	*fdhABC* (RCAP_rcc03035–03037), *fdhE* (RCAP_rcc03033)	pTrcHis A	*E. coli* MC1061	Source[29]

c. Primers used in this study

Mutation	Primer (5' to 3')	Vector	Host*
Ch2_W29A	F- caaaccgtggcggatcgttacgaggcgatg, R- gtaacgatccgccacggtttgaataccctc	pET28a	*E. coli* BL21
Ch2_Y32A	F- tgggatcgtgccgaggcgatgaagccgcag, R- catcgcctcggcacgatcccacacggtttg	pET28a	*E. coli* BL21
Ch2_F41A	F- cagtgcggtgcgggcgaaaccggtctgtgc, R- ggtttcgcccgcaccgcactgcggcttcat	pET28a	*E. coli* BL21
Ch2_F41V	F- cagtgcggtgttggcgaaaccggtctgtgc, R- ggtttcgccaacaccgcactgcggcttcat	pET28a	*E. coli* BL21
Ch2_F41L	F- cagtgcggtctgggcgaaaccggtctgtgc, R- ggtttcgcccagaccgcactgcggcttcat	pET28a	*E. coli* BL21
Ch2_F41C	F- cagtgcggttgtggcgaaaccggtctgtgc, R- ggtttcgccacaaccgcactgcggcttcat	pET28a	*E. coli* BL21
Ch2_F41Y	F- cagtgcggttatggcgaaaccggtctgtgc, R- ggtttcgccataaccgcactgcggcttcat	pET28a	*E. coli* BL21
Ch2_F43K	F- cggtttcggcaagaccggtctgtgctgccgt, R- acagaccggtcttgccgaaaccgcactgcg	pET28a	*E. coli* BL21

Ch2_F43R	F- cggtttcggccgtaccggtctgtgctgccgt, R- acagaccggtacggccgaaaccgcactgcg	pET28a	*E. coli* BL21
Ch2_T44A	F- ttcggcgaagcgggtctgtgctgccgtcac, R- gcacagacccgcttcgccgaaaccgcactgcgg	pET28a	*E. coli* BL21
Ch2_P60A	F- ccgtattaacgcgtttggcgatgagccgaaa, R- atcgccaaacgcgttaatacggcacggacc	pET28a	*E. coli* BL21
Ch2_F61A	F- attaacccggctggcgatgagccgaaagtg, R- ctcatcgccagccgggttaatacggcacgg	pET28a	*E. coli* BL21
Ch2_F154A	F- ctggcggacgcccatgagaaagataccccg, R- tttctcatgggcgtccgccagcgccgcttt	pET28a	*E. coli* BL21
Ch2_Y224A	F- gcgggttgcgccatgggcaccgacctggcg, R- ggtgcccatggcgcaacccgccaggtccgc	pET28a	*E. coli* BL21
Ch2_F234A	F- gatatcctggctggtaccccggcgccggtg, R- cggggtaccagccaggatatccgccaggtc	pET28a	*E. coli* BL21
Ch2_F386A	F- attgataccgctaaacgtcgtaagggtaaa, R- acgacgtttagcggtatcaatcgccagacg	pET28a	*E. coli* BL21
Ch2_L583A	F- cattggtgtggcgccgccgatcaccggca, R- gatcggcggcgccacaccaatgtgggtcgg	pET28a	*E. coli* BL21
Ch2_L612A	F- catcgttgaagcggacccggagaccgcggc, R- ctccgggtccgcttcaacgatgaagtagcc	pET28a	*E. coli* BL21
Ch2_W636A	F- ggtctgccggcgtgaggatccgaattcgag, R- ggatcctcacgccggcagacccagacccgc	pET28a	*E. coli* BL21
Ch2_M116A	F- ggcgagctatgcgattaaggaccgtaccaaa, R- ggtccttaatcgcatagctcgccgctttgcc	pET28a	*E. coli* BL21
Ch2_M355A	F- caccatggaagcgagcaaaatcaccggtg, R- tgattttgctcgcttccatggtggtaataacg	pET28a	*E. coli* BL21
Ch2_C344A	F- cattgcggaggcgaccggtaccaccgttat, R- tggtaccggtcgcctccgcaatggtcgcca	pET28a	*E. coli* BL21
Ch2_C496A	F- ggatgagctggcgggcgacggtctgaaagc, R- gaccgtcgcccgccagctcatccacgttcg	pET28a	*E. coli* BL21
Ch2_R57E	F- ggtccgtgcgaaattaacccgtttggcgat, R- cgggttaatttcgcacggaccttgcaggca	pET28a	*E. coli* BL21
Ch2_R57G	F- ggtccgtgcggtattaacccgtttggcgat, R- cgggttaataccgcacggaccttgcaggca	pET28a	*E. coli* BL21
Ch2_R57S	F- ggtccgtgctctattaacccgtttggcgat, R- cgggttaatagagcacggaccttgcaggca	pET28a	*E. coli* BL21
Ch2_R57F	F- ggtccgtgcttcattaacccgtttggcgat, R- cgggttaatgaagcacggaccttgcaggca	pET28a	*E. coli* BL21
Ch2_R57Q	F- ggtccgtgccaaattaacccgtttggcgat, R- cgggttaatttggcacggaccttgcaggca	pET28a	*E. coli* BL21
Ch2_R57A	F- ggtccgtgcgctattaacccgtttggcgat, R- cgggttaatagcgcacggaccttgcaggca	pET28a	*E. coli* BL21
Ch2_N59L	F- tgccgtattcttccgtttggcgatgagccg, R- gccaaacggaagaatacggcacggaccttg	pET28a	*E. coli* BL21
Ch2_N59A	F- tgccgtattgctccgtttggcgatgagccg, R- gccaaacggagcaatacggcacggaccttg	pET28a	*E. coli* BL21
Ch2_N59F	F- tgccgtatttttccgtttggcgatgagccg, R- gccaaacggaaaaatacggcacggaccttg	pET28a	*E. coli* BL21
Ch2_N59K	F- tgccgtattaagccgtttggcgatgagccg, R- gccaaacggcttaatacggcacggaccttg	pET28a	*E. coli* BL21
Ch2_N59D	F- tgccgtattgatccgtttggcgatgagccg, R- gccaaacggatcaatacggcacggaccttg	pET28a	*E. coli* BL21
Ch2_N59G	F- tgccgtattggtccgtttggcgatgagccg, R- gccaaacggaccaatacggcacggaccttg	pET28a	*E. coli* BL21
Ch2_R57G/N59L	F- tgcggtattcttccgtttggcgatgagccg, R- gccaaacggaagaataccgcacggaccttg	pET28a	*E. coli* BL21
Ch2_R57G/N59F	F- tgcggtatttttccgtttggcgatgagccg, R- gccaaacggaaaaataccgcacggaccttg	pET28a	*E. coli* BL21
Ch2_R57G/N59K	F- tgcggtattaagccgtttggcgatgagccg, R- gccaaacggcttaataccgcacggaccttg	pET28a	*E. coli* BL21
Ch2_A559W	F- gcgcggcggaatggatgcatgagaaggcggtgg, R-tctcatgcatccattccgccgcgctcgcaacc	pET28a	*E. coli* BL21
Ch2_GLW	F- tgcggtattcttccgtttggcgatgagccg, R- gccaaacggaagaataccgcacggaccttg<br>F-gcgcggcggaatggatgcatgagaaggcggtgg, R-tctcatgcatccattccgccgcgctcgcaacc	pET28a	*E. coli* BL21
Dv_F44A	F- agcctgtaaagcctgtgaattgggcaccac, R- ccaattcacaggctttacaggctggcgtct	pET28a	*E. coli* BL21
Rr_F43A	F- aatgcggcgctggctcggccggcctgtgttg, R- ccgagccagcgccgcattggggctgctgctt	pET28a	*E. coli* BL21

[*] The host *E. coli* BL21 strain contains pRKISC plasmid carrying Fe–S cluster assembly proteins.

Supplementary Table 4 | Specific activities of *Ch*CODH2 F41 variants

***Ch*CODH2 F41 variants**	**Specific activity (U·mg^{-1})**	**Relative activity (%)**
WT (F41)	1,800 ± 29	100 ± 2
F41Y	1,900 ± 68	105 ± 4
F41A	0.3 ± 0.03	0.02 ± 0.002
F41V	8.1 ± 0.9	0.5 ± 0.1
F41L	2.4 ± 0.7	0.1 ± 0.04

The enzyme reaction was performed at 30°C using CO-saturated HEPES buffer pH 8 with 20 mM EV_{ox}. The absorbance change at 578 nm was spectrophotometrically monitored in triplicate to determine the specific activity. The data represent the mean ± standard deviation (S.D.), as determined from n = 3 independent experiments. Abbreviations: *Ch*, *Carboxydothermus hydrogenoformans*; CODH, carbon monoxide dehydrogenase; EV_{ox}, oxidized ethyl viologen.

Supplementary Table 5 | F41-neighbouring residues

Category	Residues in *Ch*CODH2
**Within 4 Å at F41**	**C39–G40, G42–C47, R57–N59**
▪ Inward-facing side chain	G42, G45, I58
▪ D-cluster maintenance	C39, G40, C47
▪ Backbone interaction with F41	L46, T44
▪ Substrate tunnel-forming residues	E43
**Remaining residues**	**R57, N59**

Supplementary Table 6 | K_M for viologens in CODHs and FDHs

**Group (EC no.)**	**Name**	**Viologens***	**K_m (mM)**	**Temp. (°C)/pH**	**References**
CODH (1.2.7.4)	*Ch*CODH2	EV_{ox}	2.3 ± 0.1	30°C/pH 8	This study
		MV_{ox}	4	20°C/pH 9.5	Source[30]
	*Ch*CODH4	EV_{ox}	1.3 ± 0.1	30°C/pH 8	This study
	*Mb*CODH	MV_{ox}	7.1 ± 0.01	25°C/pH 7	Source[6]
	*Mt*CODH	MV_{ox}	3.0 ± 0.01	50°C/pH 8.4	Source[31]
	*To*CODH	EV_{ox}	2.4 ± 0.2	30°C/pH 8	This study
FDH (1.17.1.9)	*Me*FDH1	EV_{red}	0.03 ± 0.002	30°C/pH 7	This study
	*Rc*FDH	EV_{red}	0.01 ± 0.001	30°C/pH 7	This study

* The measurement of CODH activity employed EV_{ox} because CO oxidation activity necessitates an oxidized viologen to receive electrons from the reaction. Values are the means ± standard variation, $n = 3$.

Abbreviations: CODH, carbon monoxide dehydrogenase; FDH, formate dehydrogenase; ox, oxidized; red, reduced; EV, ethyl viologen; MV, methyl viologen, *Ch*, *Carboxydothermus hydrogenoformans; Mb, Methanosarcina barkeri, Me, Methylobacterium extorquens; Rc, Mt, Moorella thermoacetica, Rhodobacter capsulatus; To, Thermococcus onnurineus.*

Supplementary Table 7 | Kinetic constants of *Ch*CODH2 R57 and N59 variants

Enzyme	Specific activity[a] ($U \cdot mg^{-1}$)	K_M^{EV} (mM)	k_{cat}^{EV} (s^{-1})	k_{cat}/K_M^{EV} ($s^{-1} \cdot mM^{-1}$)
Wild type				
*Ch*CODH2	1,800 ± 29	2.3 ± 0.1	2,200 ± 28	1,000 ± 11
***Ch*CODH2 R57 variants**				
R57A	3,400 ± 82	0.5 ± 0.1	3,400 ± 78	6,700 ± 330
R57E	3,300 ± 56	0.4 ± 0.1	3,400 ± 13	9,000 ± 153
R57F	1,700 ± 28	0.5 ± 0.1	1,800 ± 23	3,500 ± 231
R57G	3,800 ± 29	0.3 ± 0.1	4,200 ± 51	14,700 ± 576
R57Q	3,600 ± 45	0.4 ± 0.1	3,600 ± 27	8,600 ± 130
R57S	3,500 ± 49	0.4 ± 0.1	3,500 ± 86	9,700 ± 621
***Ch*CODH2 N59 variants**				
N59A	5,800 ± 29	1.0 ± 0.1	5,600 ± 18	5,400 ± 165
N59D	5,200 ± 28	0.5 ± 0.1	4,500 ± 4	9,000 ± 82
N59F	1,600 ± 20	1.8 ± 0.2	1,400 ± 74	800 ± 63
N59G	5,100 ± 30	0.8 ± 0.1	4,800 ± 57	6,100 ± 97
N59K	2,600 ± 25	3.3 ± 0.1	1,900 ± 50	590 ± 23
N59L	3,400 ± 19	1.6 ± 0.1	3,100 ± 43	1,900 ± 56

[a] Specific activities were determined at 20 mM ethyl viologen (EV) in HEPES buffer saturated with CO (30°C, pH 8). Values are the means ± standard variation, n = 3.

[*] Kinetic data were assayed at 30°C, pH 8. The kinetic parameters were calculated by fitting the initial rates obtained at six different EV concentrations (0.0625–32 mM) to the nonlinear hyperbolic regression using SigmaPlot 10.0. All enzymatic activities were determined in triplicate (see details in the Methods section).

[†] The values of k_{cat} were calculated from V_{max} for EV.

Supplementary Table 8 | Gas compositions of industrial off-gas and waste plastic syngas

Provider		Coke oven gas (COG)	Blast furnace gas (BFG)	Lintz–Donawitz gas (LDG)	Solid refuse fuel (SRF)-derived gas
		Hyundai Steel	Hyundai Steel	Hyundai Steel	Korea Institute of Energy Research
Component (%)	**CO**	6.2 ± 0.1	25.5 ± 0.3	49.4 ± 1.9	7.7 ± 0.9
	**H_2**	55.4 ± 0.5	3.8 ± 0.2	1.4 ± 0.4	2.6 ± 0.6
	**N_2**	8.6 ± 0.6	46.7 ± 0.1	29.2 ± 1.9	38.5 ± 7.4
	**CO_2**	2.4 ± 0.1	23.6 ± 0.2	19.3 ± 0.6	42.8 ± 3.1
	**CH_4**	24.2 ± 0.4	–	–	4.5 ± 1.4
	**C_mH_n**	3.1 ± 0.1	–	–	4.0 ± 1.3
	**O_2**	0.1 ± 0.02	0.01 ± 0.00	0.01 ± 0.02	–

[*] Compositions of industrial off-gases are from the previous study[26].

[†] Solid refuse fuel (SRF) derived from the gasification of mixed plastic waste was provided by the Korea Institute of Energy Research[32,33].

Supplementary Table 9 | Statistics for data collection and refinement

Data set	R57G/N59L apo (PDB ID: 8X9D)	R57G/N59L apo in 5% PEG (PDB ID: 8X9E)	R57G/N59L with EV (PDB ID: 8X9F)	R57G/N59L with BV (PDB ID: 8X9G)	F41C (PDB ID: 8X9H)
A. Data collection					
Energy (keV)	12.398	12.398	12.398	12.398	7.140
Space group	*C*2	*C*2	*C*2	*C*2	*C*2
Cell dimensions					
a, *b*, *c* (Å)	112.0, 74.6, 71.3	112.1, 75.1, 71.1	112.3, 74.8, 71.2	112.6, 75.4, 72.2	112.1, 75.3, 70.7
α, β, γ (°)	90.0, 111.5, 90.0	90.0, 111.4, 90.0	90.0, 111.2, 90.0	90.0, 111.8, 90.0	90.0, 111.1, 90.0
Resolution range (Å)	50.00 – 2.10 (2.14 – 2.10)*	50.00 – 2.50 (2.54 – 2.50)*	30.00 – 2.50 (2.54 – 2.50)*	50.00 – 3.10 (3.15 – 3.10)*	40.78 – 2.20 (2.21 – 2.20)*
Total/ unique reflections	106,342 / 31,368	71,132 / 18,938	69,338 / 19,005	51,029 / 10,065	112,785 / 27,608
Completeness (%)	99.5 (99.4)*	99.9 (92.8)*	98.0 (98.9)*	98.8 (98.8)*	98.4 (96.8)*
Average I / σI (I)	14.4 (2.4)*	8.7 (1.4)*	8.4 (1.9)*	9.6 (2.6)*	19.07 (13.0)*
$CC_{1/2}$	98.9 (82.1)*	99.5 (81.3)*	98.6 (80.9)*	97.4 (87.3)*	99.6 (99.1)*
R_{merge}† (%)	8.0 (47.8)*	9.9 (47.4)*	11.4 (43.9)*	14.8 (44.3)*	5.6 (7.8)*
Redundancy	3.4 (3.4)*	3.8 (3.4)*	3.6 (3.7)*	5.1 (4.7)*	4.1 (3.9)*
B. Model refinement statics					
Resolution range (Å)	33.02 – 2.11	35.22 – 2.50	28.05 – 2.48	35.32 – 3.11	37.70 – 2.20
R_{work} / R_{free}‡ (%)	16.53 / 21.91	17.66 / 22.69	24.46 / 26.73	22.92 / 27.23	14.11 / 19.31
No. atoms					
Protein	4,682	4,661	4,646	4,646	4,648
Cluster atoms	22	22	21	21	22
EV/BV molecules			16	26	
Water	327	119	117	3	355
B-factors (Å^2)					
Protein	28.75	40.82	37.19	56.27	21.82
Cluster atoms	23.1	38.26	36.50	52.53	24.80
EV/BV molecules			50.71	74.74	
Water	32.64	39.38	35.61	48.27	27.00
R.m.s. deviations					
Bond lengths (Å)	0.009	0.010	0.003	0.003	0.008
Bond angles (°)	1.063	1.145	0.628	0.712	0.979
Ramachandran					
Favored (%)	96.67	96.20	96.99	96.51	97.31
Allowed (%)	3.17	3.65	3.01	3.33	2.38
Outliers (%)	0.16	0.16	0.00	0.16	0.32

* Values in parentheses refer to the highest-resolution shell. Data were collected from one crystal.

† $R_{merge} = \Sigma_{hkl}\Sigma_i \mid I_i(hkl) - \langle I(hkl)\rangle \mid / \Sigma_{hkl}\Sigma_i I_i(hkl)_i$, where $I(hkl)$ is the intensity of reflection *hkl*, Σ_{hkl} is the sum over all reflections, and Σ_i is the sum over i measurements of reflection *hkl*.

‡ $R = \Sigma_{hkl} \mid |F_{obs}| - |F_{calc}| \mid / \Sigma_{hkl} |F_{obs}|$, where R_{free} was calculated for a randomly chosen 10% of reflections, which were not used for structural refinement, and R_{work} was calculated for the remaining ones.

Supplementary Table 10 | Data collection statistics for Fe anomalous data

Data set	R57G/N59L apo (PDB: 8X9D)	F41C (PDB: 8X9H)
Data collection		
Energy (keV)	7.140	7.140
Space group	*C*2	*C*2
Unit-cell length (*a*, *b*, *c*, Å)	112.6, 75.2, 71.6	112.1, 75.3, 70.7
Unit-cell angle (α, β, γ, °)	90.0 111.6, 90.0	90.0, 111.1, 90.0
Resolution range (Å)	50.00 – 2.80 (2.82 – 2.80)*	50.0 – 1.80 (1.81 – 1.80)*
Total / unique reflections	55,136 / 25,928	202,769 / 94,544
Completeness (%)	96.3 (96.6)*	94.3 (90.2)*
Average $I/\sigma(I)$	18.7 (9.6)*	15.4 (8.6)*
$CC_{1/2}$	99.8 (98.9)*	99.5 (98.7)*
$R_{merge}^{\dagger}$ (%)	3.4 (6.8)*	4.2 (7.2)*
Redundancy	2.1 (2.1)*	2.1 (2.1)*

*Values in parentheses refer to the highest-resolution shell. Data were collected from one crystal.

† $R_{merge} = \Sigma_{hkl}\Sigma_i \mid I_i(hkl) - \langle I(hkl)\rangle \mid / \Sigma_{hkl}\Sigma_i I_i(hkl)_i$, where $I(hkl)$ is the intensity of reflection *hkl*, Σ_{hkl} is the sum over all reflections, and Σ_i is the sum over i measurements of reflection *hkl*.

Supplementary Figures 1–19

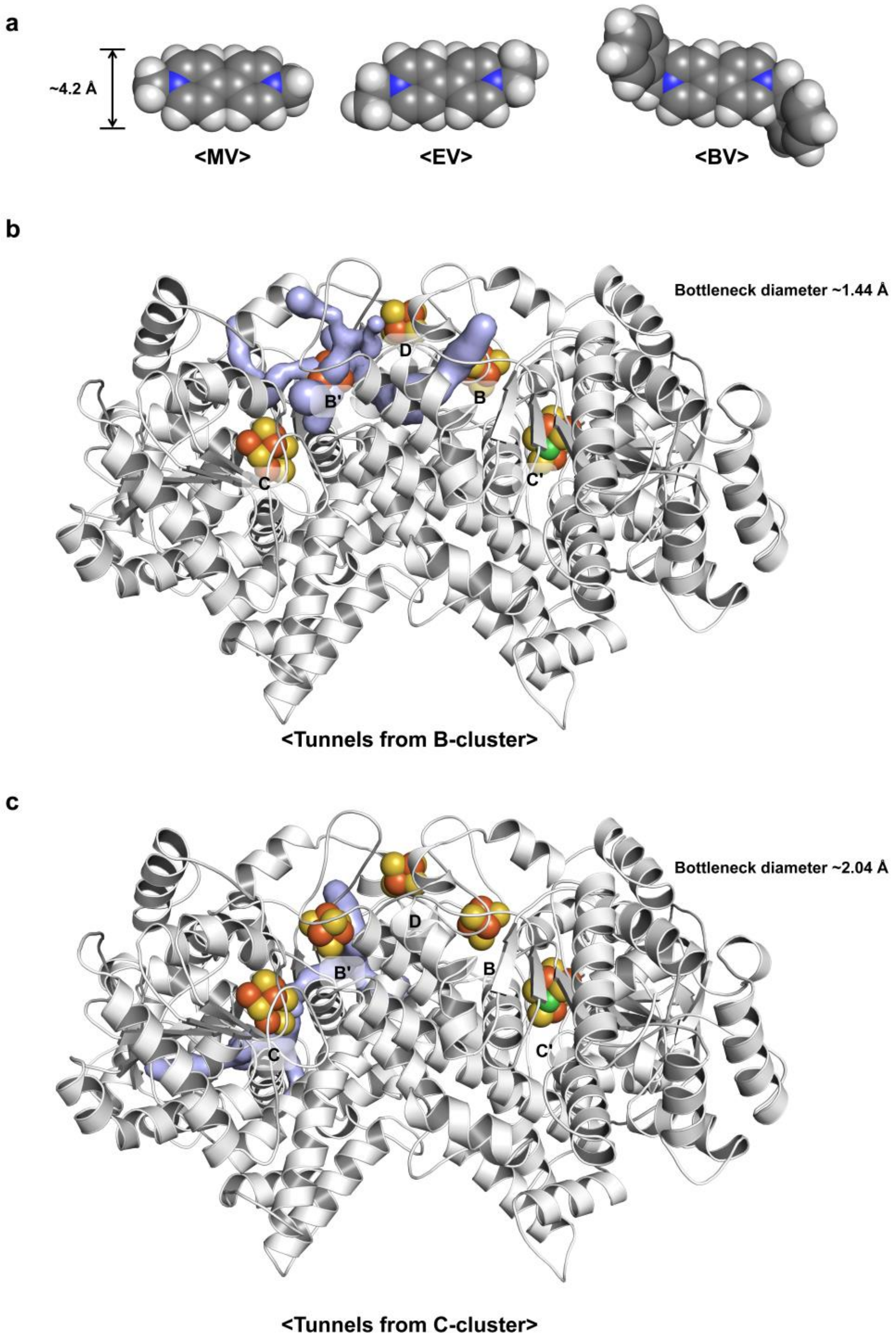

Supplementary Figure 1 | Viologens' inability to traverse substrate tunnels due to size constraints. a, The van der Waals diameter of viologens (MV, methyl viologen; EV, ethyl viologen; BV, benzyl viologen). **b,** Bottleneck diameter of substrate tunnels derived from B-cluster in *Ch*CODH2. **c,** The bottleneck diameter of substrate tunnels from catalytic C-cluster in *Ch*CODH2. The substrate were tunnels predicted as light blue through CAVER analysis[34].

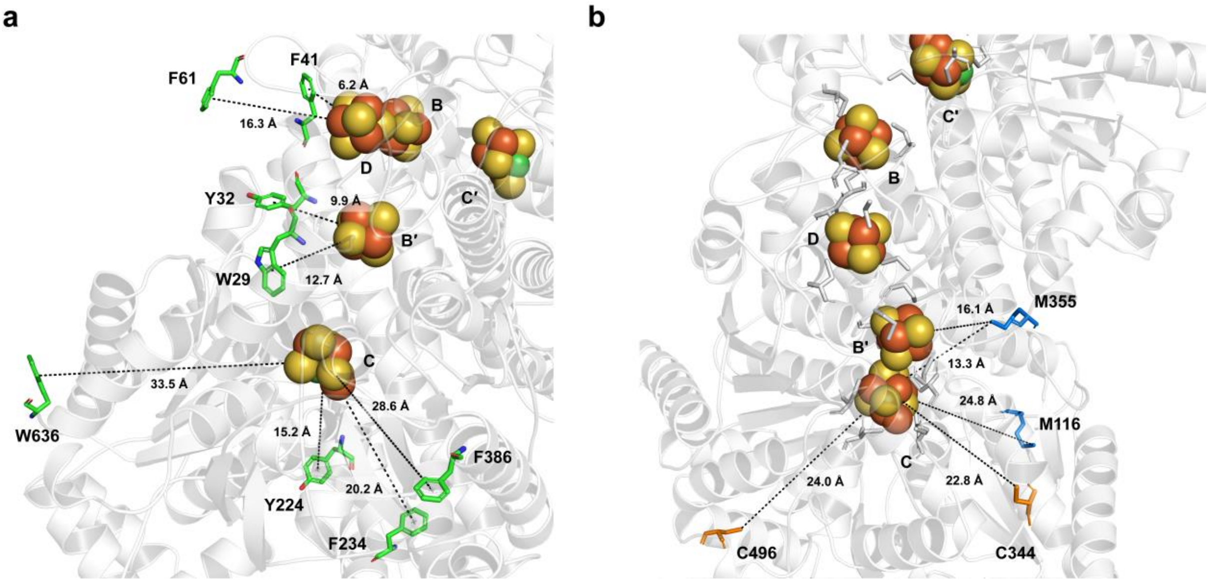

Supplementary Figure 2 | Surface-exposed aromatic and sulfur-containing residues in *Ch*CODH2. a, Distance between surface aromatic residues (F/Y/W) and Fe–S cluster. **b,** Distance between surface sulfur-containing residues (C/M) and Fe-S cluster. The cysteine residues interacting with Fe-S cluster were depicted in grey sticks, including C48, C51, C56, and C70 in the B-cluster; C295, C295, C333, C446, C476, and C526 in the C-cluster; and C39 and C47 in the D-cluster.

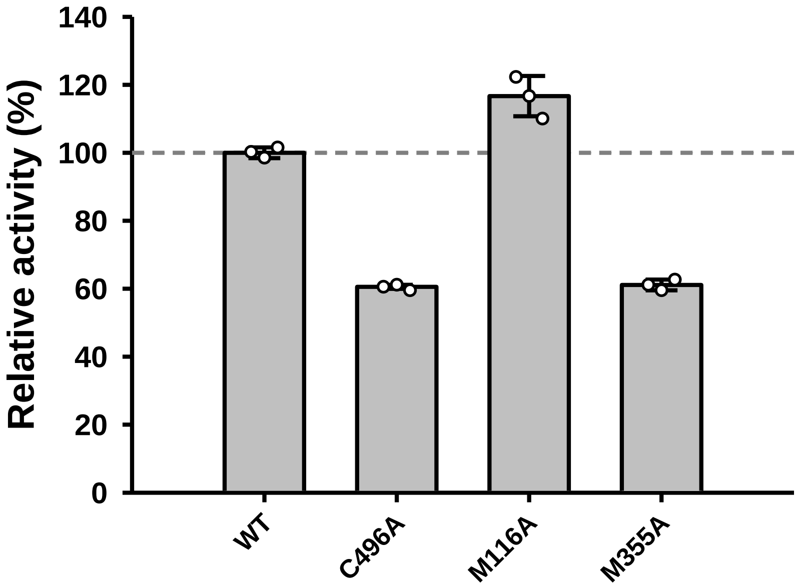

Supplementary Figure 3 | Relative activities of C496A, M116A and M355A variants. Enzymatic activity is anaerobically measured through CO oxidation at 30°C in CO-saturated HEPES buffer pH 8 containing 20 mM EV_{ox}. The relative activities of variants were calculated by comparison of the WT's specific activity. White dots for each point overlay bar charts. The data represent the mean ± S.D., determined from n = 3 independent experiments. C344A was not expressed in *E. coli*.

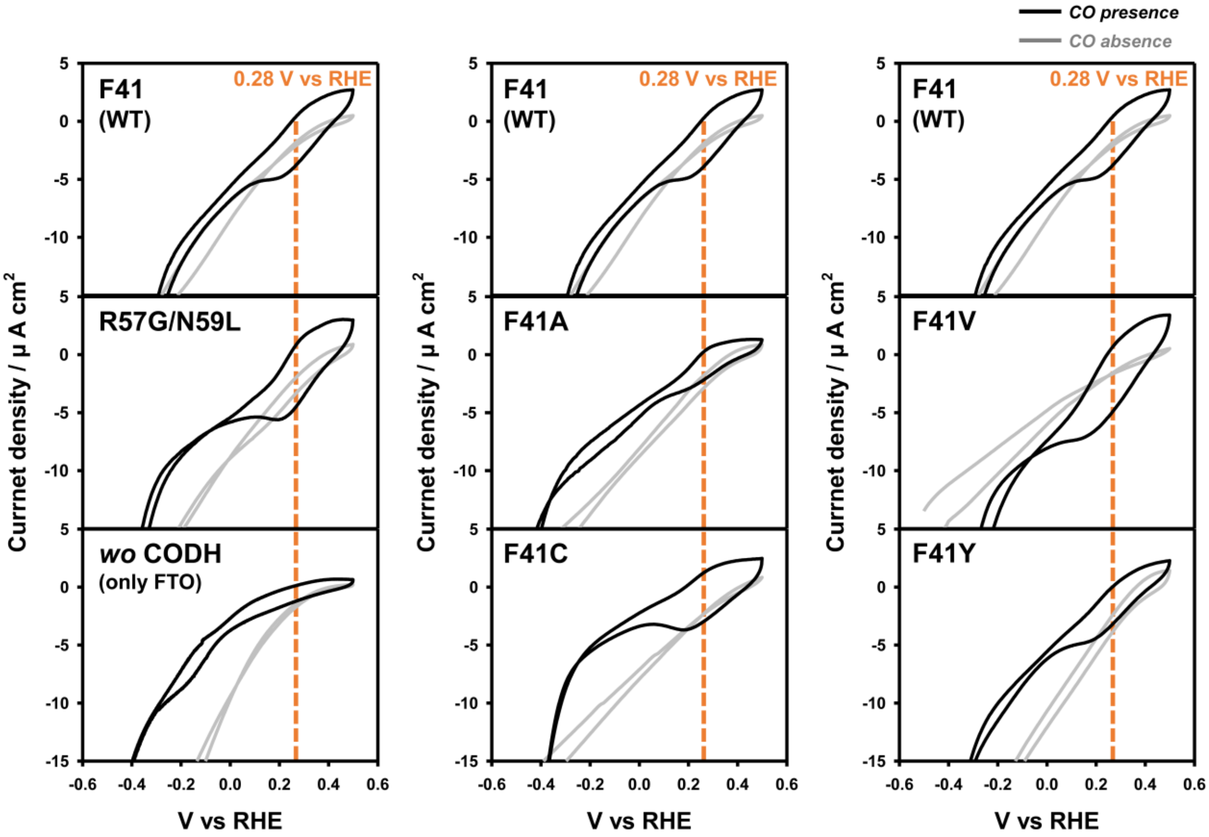

Supplementary Figure 4 | Viologen-free electrochemical reaction of *Ch*CODH2 variants. Cyclic voltammetry (CV) was conducted on fluorine-tin oxide (FTO) electrodes using CODH enzyme films. The black lines represent experiments performed with 100% (*v*/*v*) CO, while the grey lines indicate experiments carried out without CO. Experimental conditions included a temperature of 25°C, a 200 mM HEPES/NaOH buffer (pH 8.0), and a scan rate of 10 mV s^{-1}. The observed potential (V vs RHE) in CO oxidation reactions is indicated by an orange dotted line at 0.28V vs RHE. All measurements were conducted in triplicate to verify reproducible CV profiles. Abbreviations: CODH, carbon monoxide dehydrogenase; FTO, fluorine-tin oxide; RHE, reversible hydrogen electrode; *wo*, without.

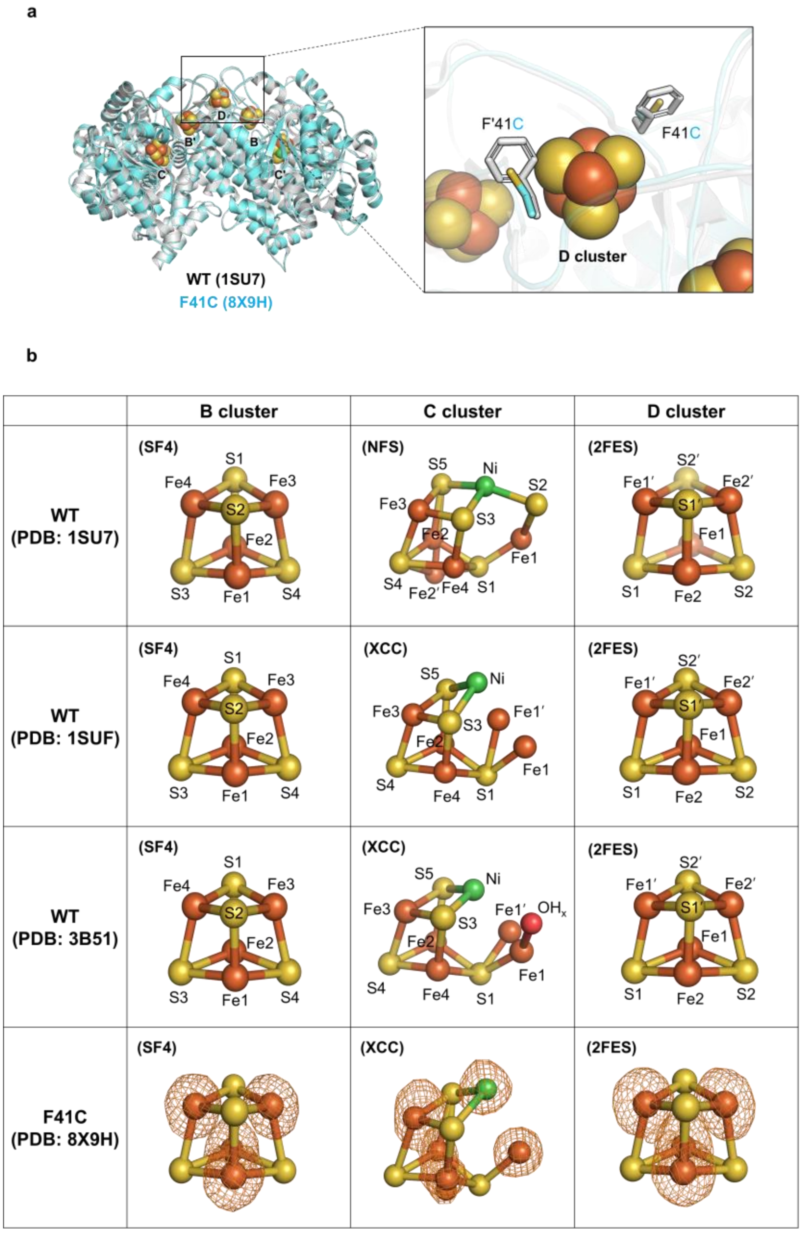

Supplementary Figure 5 | F41C structure and Fe anomalous maps of clusters. a, Structural comparison of the D-cluster region in the crystal structures of *Ch*CODH2 WT (grey, PDB ID: 1SU7)[21] and F41C variant (light blue, PDB ID: 8X9H). **b,** Anomalous difference Fourier maps illustrating the positions of Fe atoms in B, C, and D clusters contoured at 10, 5, and 10 σ, respectively, are shown in orange mesh. Fe, S, and Ni atoms are coloured orange, yellow and green, respectively. For the C cluster, 1SU7[21] and 3B51[35] exhibit the Ni-4Fe-4S-mut2S (NFS) and Ni-4Fe-4S-OH_x (XCC with OH_x) conformations, respectively, 1SUF[21] and 8X9H display the Ni-4Fe-4S (XCC) conformation. Both the B and D clusters maintain identical conformations across all structure. In F41C, despite a *Fo-Fc* difference map (3 σ) indicating a potential site next to Fe1, the S2 atom and the OH_x ligand were not modelled within the C cluster due to indeterminate electron density. Consequently, the XCC model, which lacks the μ_2-S ligand (S2) and OH_x, ligand[35,36], was found to be a suitable and accurate model for this variant's conformation.

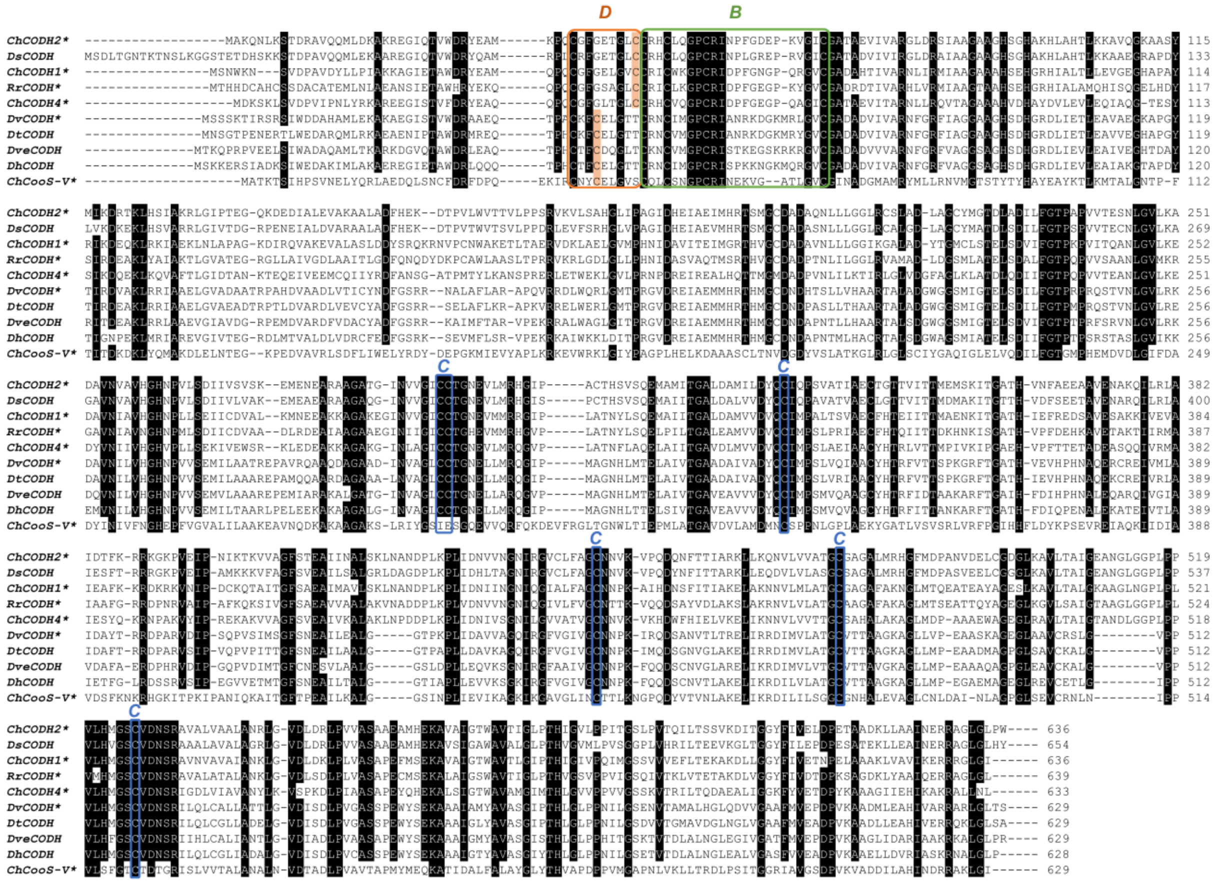

Supplementary Figure 6 | Multiple alignments of *Ch*CODH-related sequences. In the amino acid alignments of *Ch*CODHs, the amino acid residues are displayed in black shading with white letters (identical residue, >90% threshold for shading). The characterized CODHs are marked with asterisks (*). The boxes indicate cysteine residues coordinating each Fe–S cluster (B, C, D): C48, C51, C56, and C70 for B-cluster; C294–295, C333, C446, and C476 for C-cluster; C39, C47, C'39, and C'47 for D-cluster. The CODHs are as follows: *Ch*CODH1 (WP_011344718), *Ch*CODH2 (WP_011343033), *Ch*CODH4 (WP_011343666), *Ch*CooS-V (WP_011342982) from *Carboxydothermus hydrogenoformans*; *Ds*CODH from *Desulfofundulus salinum* (WP_121452276); *Rr*CODH from *Rhodospirillum rubrum* (WP_011389181); *Dv*CODH from *Desulfovibrio vulgaris* (WP_010939375); *Dt*CODH from *D. termitidis* (WP_035067836); *Dve*CODH from *Desulfocurvus vexinensis* (WP_028588641); *Dh*CODH from *Desulfofaba hansenii* (WP_100393885).

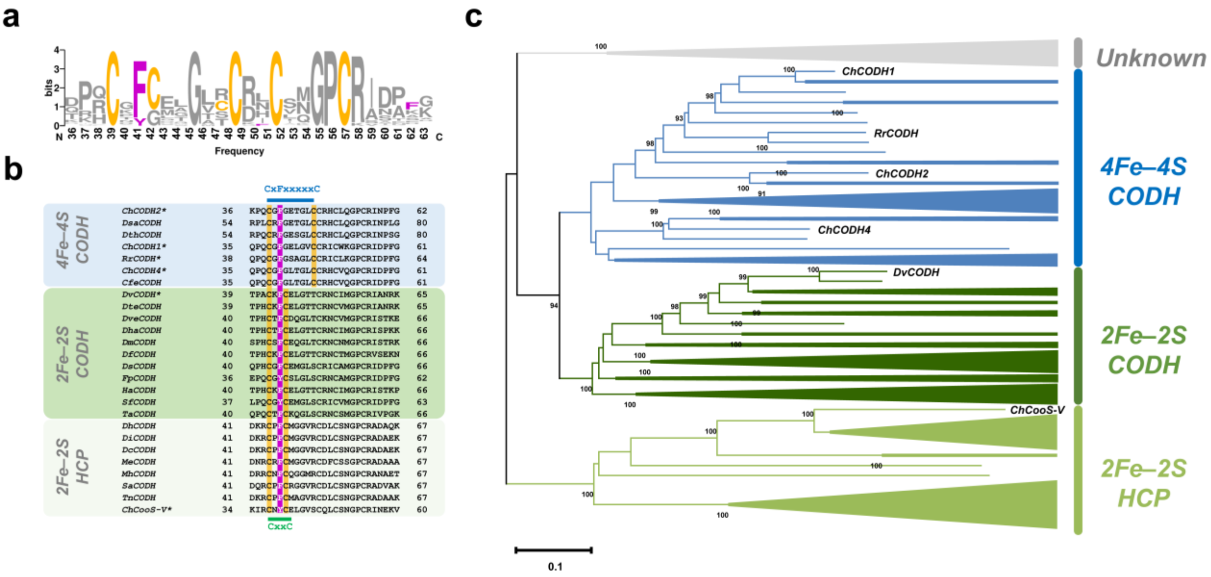

Supplementary Figure 7 | Coordinating amino acid sequences in proximity to the D-cluster. a, Sequence logo representing the frequency in the region 36–63 of *Ch*CODH2 sequences. The sequence logo was generated using WebLogo (https://weblogo.berkeley.edu/logo.cgi)[37]. **b,** Partial alignments of CODHs and HCPs (hybrid cluster proteins). The boxes indicate cysteine (orange) and phenylalanine (magenta) residues that coordinate D-cluster. The asterisks denote the CODHs that have been characterized. **c,** Phylogenetic tree of CODHs. The phylogenetic trees showed orthologous relationships based on the amino acid sequences of the CODH proteins (see Methods for details). The CODHs are as follows: *Ch*CODH1 (WP_011344718), *Ch*CODH2 (WP_011343033), *Ch*CODH4 (WP_011343666) from *Carboxydothermus hydrogenoformans*; *Cfe*CODH from *C. ferrireducens* (WP_028051453); *Dsa*CODH from *Desulfofundulus salinum* (WP_121452276); *Dth*CODH from *D. thermocisternus* (WP_084327079); *Rru*CODH from *Rhodospirillum rubrum* (WP_011389181); *Dv*CODH from *Desulfovibrio vulgaris* (WP_010939375); *Dte*CODH from *D. termitidis* (WP_035067836); *Dve*CODH from *Desulfocurvus vexinensis* (WP_028588641); *Dha*CODH from *Desulfofaba hansenii* (WP_100393885); *Dm*CODH from *Desulfobulbus mediterraneus* (WP_028583186); *Df*CODH from *Desulfoplanes formicivorans* (WP_069856808); *Ds*CODH from *Dethiosulfatarculus sandiegensis* (WP_044349155); *Fp*CODH from *Ferroglobus placidus* (WP_083777753); *Ha*CODH from *Halodesulfovibrio aestuarii* (WP_027361784); *Sf*CODH from *Syntrophobacter fumaroxidans* (WP_011699717); *Ta*CODH from *Thermodesulfobium acidiphilum* (WP_108307686); *Dh*CODH from *Desulfacinum hydrothermale* (WP_084057374); *Di*CODH from *Desulfacinum infernum* (WP_073036188); *Dc*CODH from *Desulfosoma caldarium* (WP_123291211); *Me*CODH from *Methanofollis ethanolicus* (WP_067052819); *Mh*CODH from *Methanomethylovorans hollandica* (WP_015324787); *Sa*CODH from *Syntrophorhabdus aromaticivorans* (WP_028894724); *Tn*CODH from *Thermodesulforhabdus norvegica* (WP_093393560).

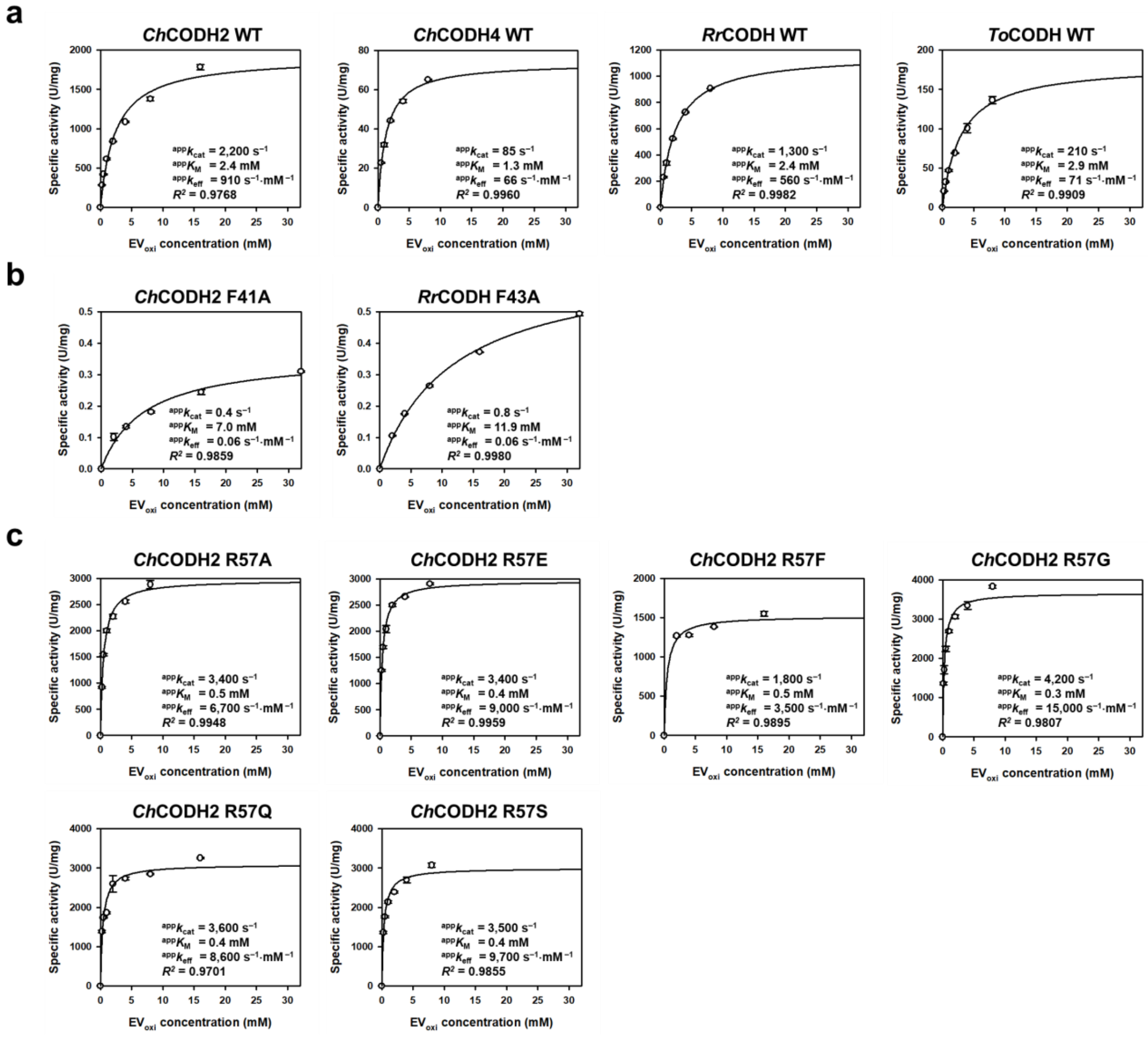

Supplementary Figure 8 | Nonlinear hyperbolic kinetic profiles of CODH wild types and selected variants. Catalytic properties of *Ch*CODH2 mutants for EV were estimated from the non-linear regression method. **a,** CODH WTs. **b,** *Ch*CODH2 F41A and *Rr*CODH F43A. **c,** *Ch*CODH2 R57 mutants. The values of ${}^{app}k_{cat}$ were calculated from V_{max} for EV. The data represent the mean ± S.D. determined from $n = 3$ independent experiments.

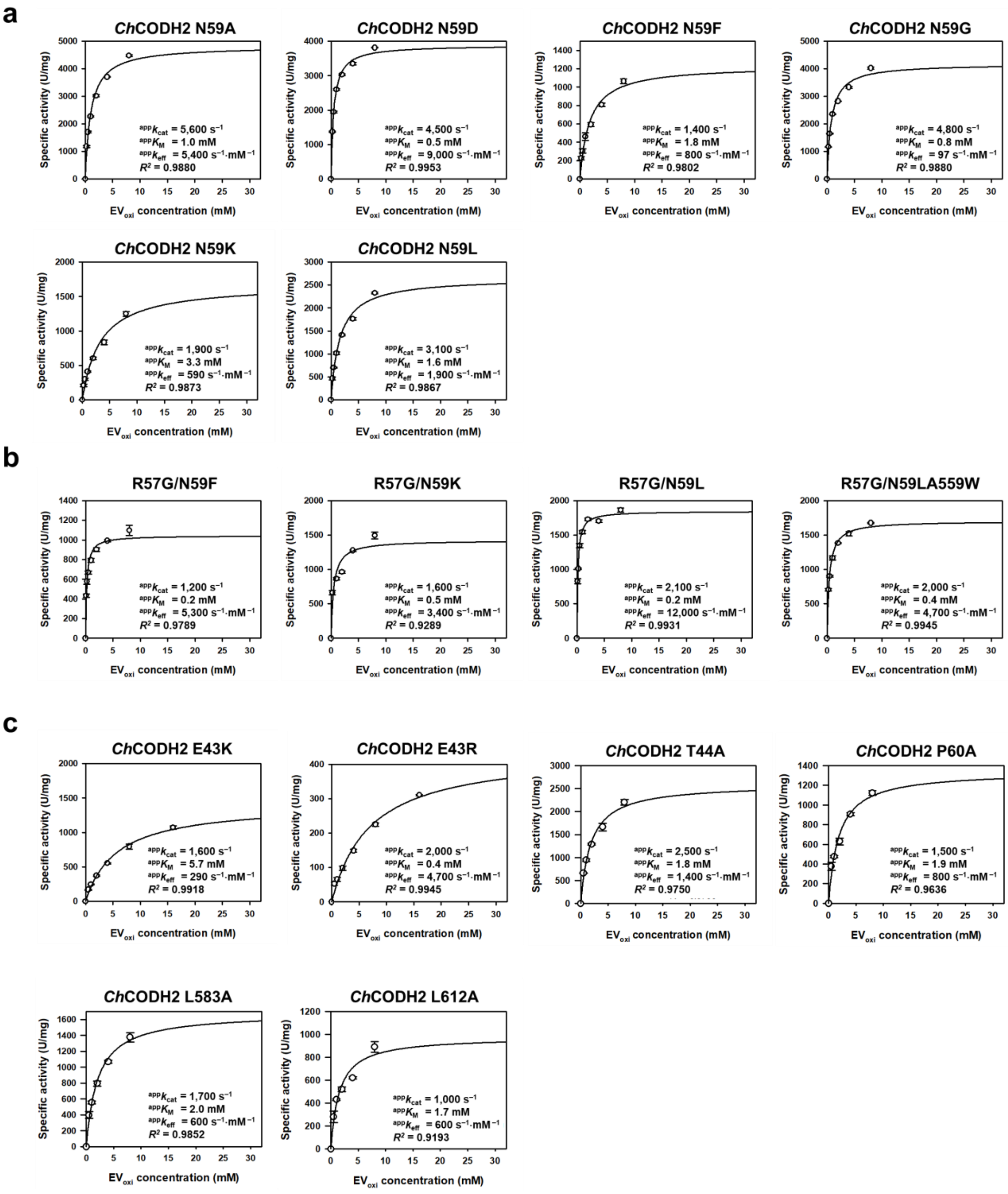

Supplementary Figure 9 | Nonlinear hyperbolic kinetic profiles across *Ch*CODH2 variants. Catalytic properties of *Ch*CODH2 mutants for EV were estimated from the non-linear regression method. **a,** *Ch*CODH2 N59 mutants. **b,** *Ch*CODH2 double and triple mutants. **c,** *Ch*CODH2 viologen-related mutants. The values of ${}^{app}k_{cat}$ were calculated from V_{max} for EV. The data represent the mean ± S.D. determined from $n = 3$ independent experiments.

a

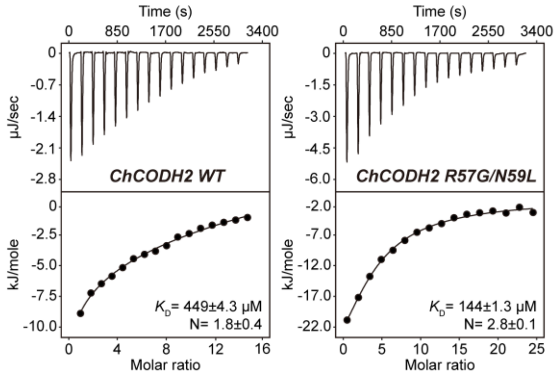

b

*Ch*CODH2	Substrate	Mediator	Binding affinity, K_D (μM)	Number of binding sites
WT	CO	Ev_{ox}	449 ± 4.3	1.8 ± 0.4
R57G/N59L	CO	EV_{ox}	144 ± 1.3	2.8 ± 0.1

Supplementary Figure 10 | ITC analysis for EV_{ox} binding in *Ch*CODH2 WT and R57G/N59L. a, Thermograms and binding isotherms depict the interaction of *Ch*CODH2 WT and R57G/N59L for oxidized ethyl viologen (EV_{ox}) in the presence of CO. **b,** Comparative analysis of binding affinity and the number of binding sites in *Ch*CODH2 WT and R57G/N59L variant. The number of binding sites indicates the average EV_{ox} molecules bound per each dimeric CODH enzyme. ITC experiments were conducted under anaerobic conditions in a customized glove box to eliminate oxygen interference. The experiments involved a concentration of 52 μM purified CODH enzyme and 2.4 μL of 2.5 mM EV_{ox}, reacted in CO-saturated buffer (20 mM Tris/HCl, pH 7.5) containing 1 mM tris(2-carboxyethyl)phosphine at 25°C (see Methods section). Values are the means ± standard variation, n = 2.

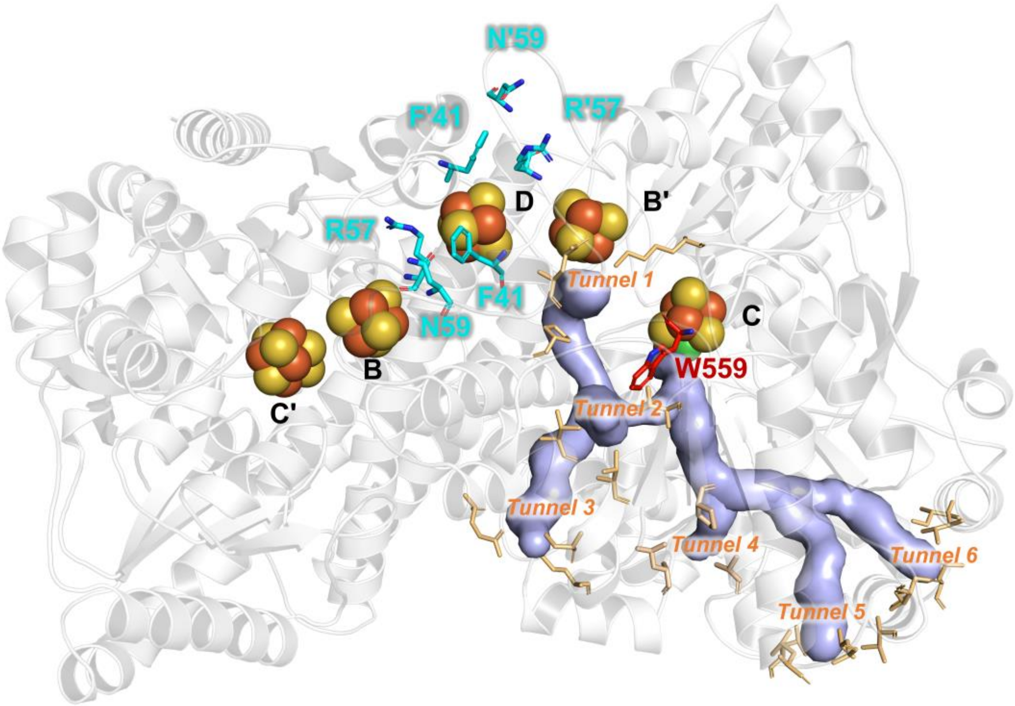

Supplementary Figure 11 | F41 neighboring and tunnel-forming residues in *Ch*CODH2 W559. The locations of residues, including R57, N59, and W559, were displayed with tunnels of the *Ch*CODH2 variant (PDB ID: 7XDM)[26]. F41, R57, and N59 (cyan stick) were found to be distantly located from W559 (red stick) and were also observed to be separated from the protein substrate tunnel exits. The residues at the tunnel exits are as follows: for tunnel #1, E43/K450/P585; for tunnel #2, T593/T597/V610; for tunnel #3, Q206/S599/I603; for tunnel #4, L168/A537/P577; for tunnel #5, V433/L543/L632/L634; for tunnel #6, L419/N431/V503/I507.

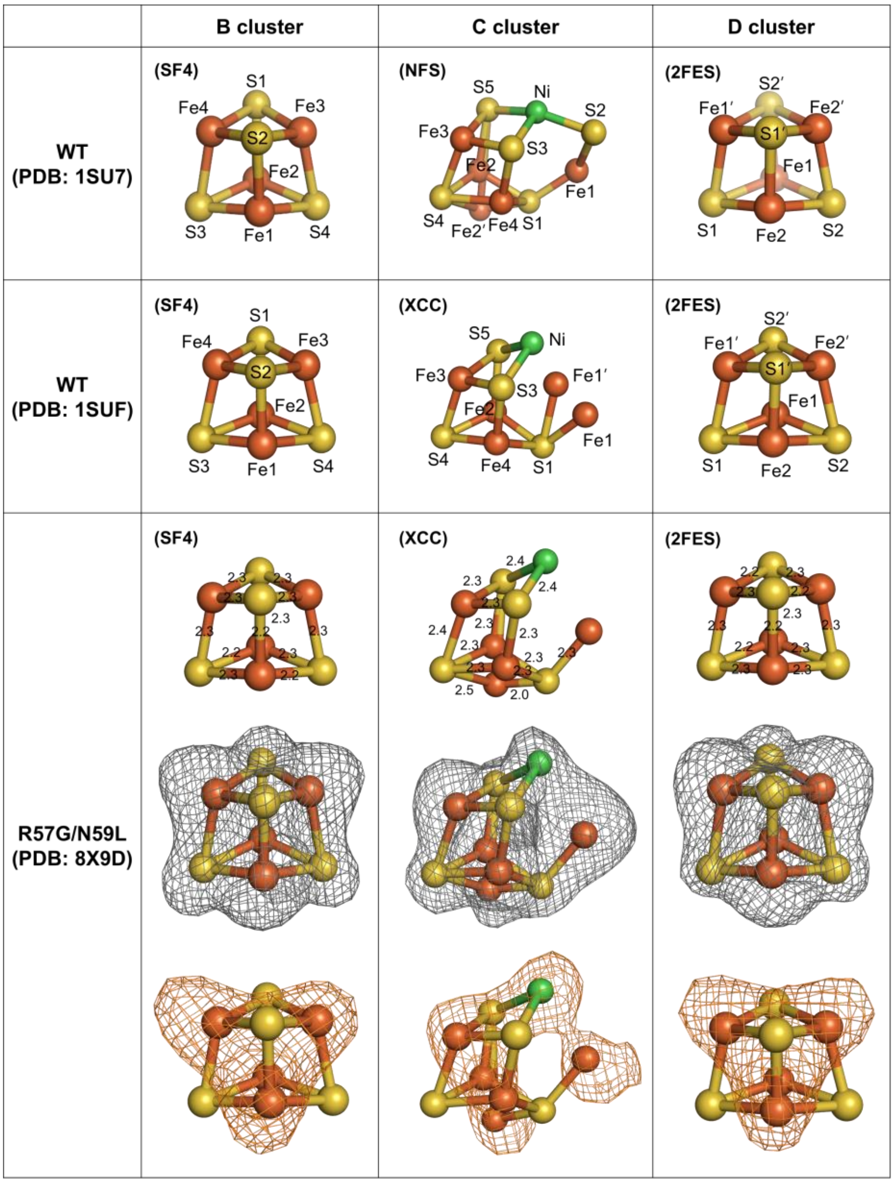

Supplementary Figure 12 | *Fo-Fc* difference electron density maps and Fe anomalous maps of clusters in R57G/N59L variant. The *Fo-Fc* difference electron-density maps of B, C, and D clusters in R57G/N59L variant are shown in grey mesh contoured at 3 σ. Fe, S, and Ni atoms are coloured orange, yellow and green, respectively. In the C cluster, 1SU7[21] adopts the Ni-4Fe-4S-mut2S (NFS) conformation, while both 1SUF[21] and 8X9H (Supplementary Figure 5) feature the Ni-4Fe-4S (XCC) conformation. The B and C clusters adopt the same conformations in both structures. In R57G/N59L, the S2 atom in the C cluster was not observed, and the XCC model, which lacks the μ_2-S ligand (S2)[35,36], was found to be a more suitable and accurate model for this conformation. Bond lengths of clusters in R57G/N59L variant are presented on each bond. Anomalous difference Fourier maps illustrating the positions of Fe atoms in clusters are shown as orange mesh, contoured at 10, 5, and 10 σ in each cluster.

a

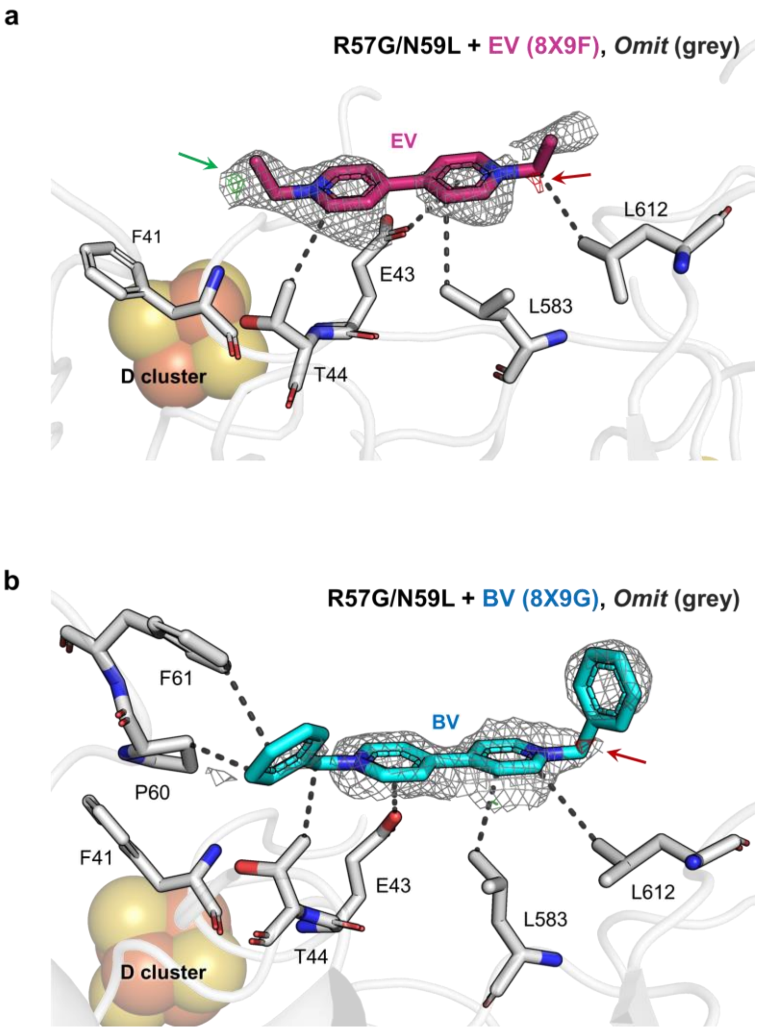

Supplementary Figure 13 | *Omit* maps of viologens in the R57G/N59L variants. a, The *omit* map of EV in the R57G/N59L variant is displayed in grey mesh contoured at 2 σ. Accompanying this is the *Fo-Fc* difference electron-density map at 2.5 σ, with positive and negative levels shown in green and red, respectively, indicated by corresponding colored arrows. The black dashed lines indicate electrostatic and hydrophobic interactions between EV, BV, and surrounding residues. Interacting residues with viologens are represented in stick form. **b**, For BV in the R57G/N59L variant, the *omit* map is similarly shown in grey mesh contoured at 2 σ, along with the *Fo-Fc* difference electron-density maps at 2.5 σ in green and red for positive and negative levels, respectively.

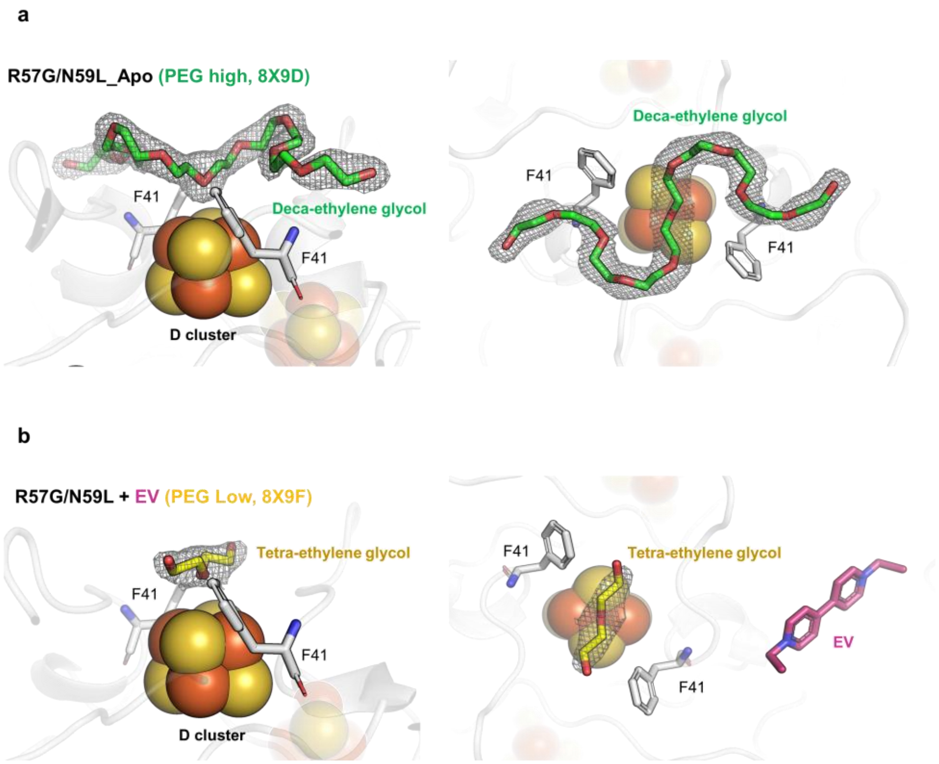

Supplementary Figure 14 | PEG Electron Density in R57G/N59L Variants. a, 2*Fo-Fc* maps of deca-ethylene glycol in R57G/N59L high PEG structure (PDB: 8X9D), contoured at 1 σ (grey mesh). **b**, *2Fo-Fc* maps of tetra-ethylene glycol in R57G/N59L low PEG structure with EV complex (PDB: 8X9F), contoured at 1 σ (grey mesh).

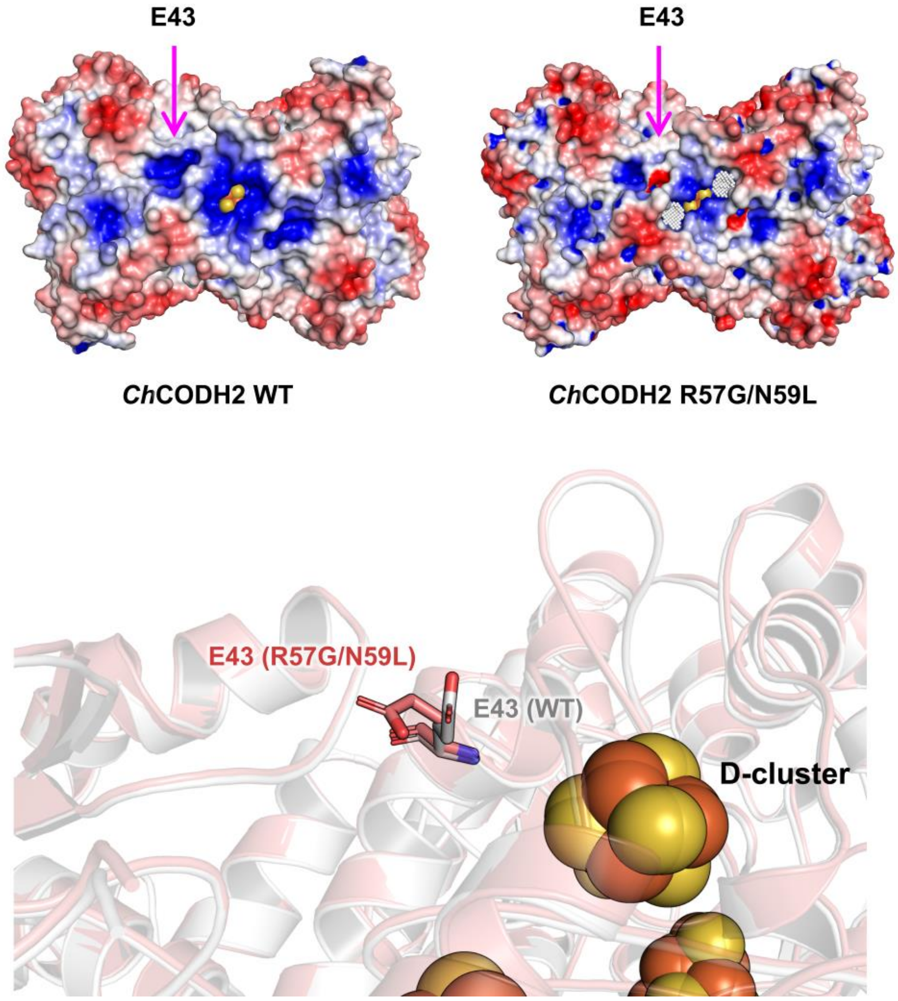

Supplementary Figure 15 | Structural changes in Glu43 residues and surface charge differences in *Ch*CODH2 WT and R57G/N59L. The observed changes in surface charge between the WT and R57G/N59L structures are attributed to differences in the torsion of E43 residue side-chains. These alterations are anticipated to significantly influence viologen binding in both WT and R57G/N59L variants.

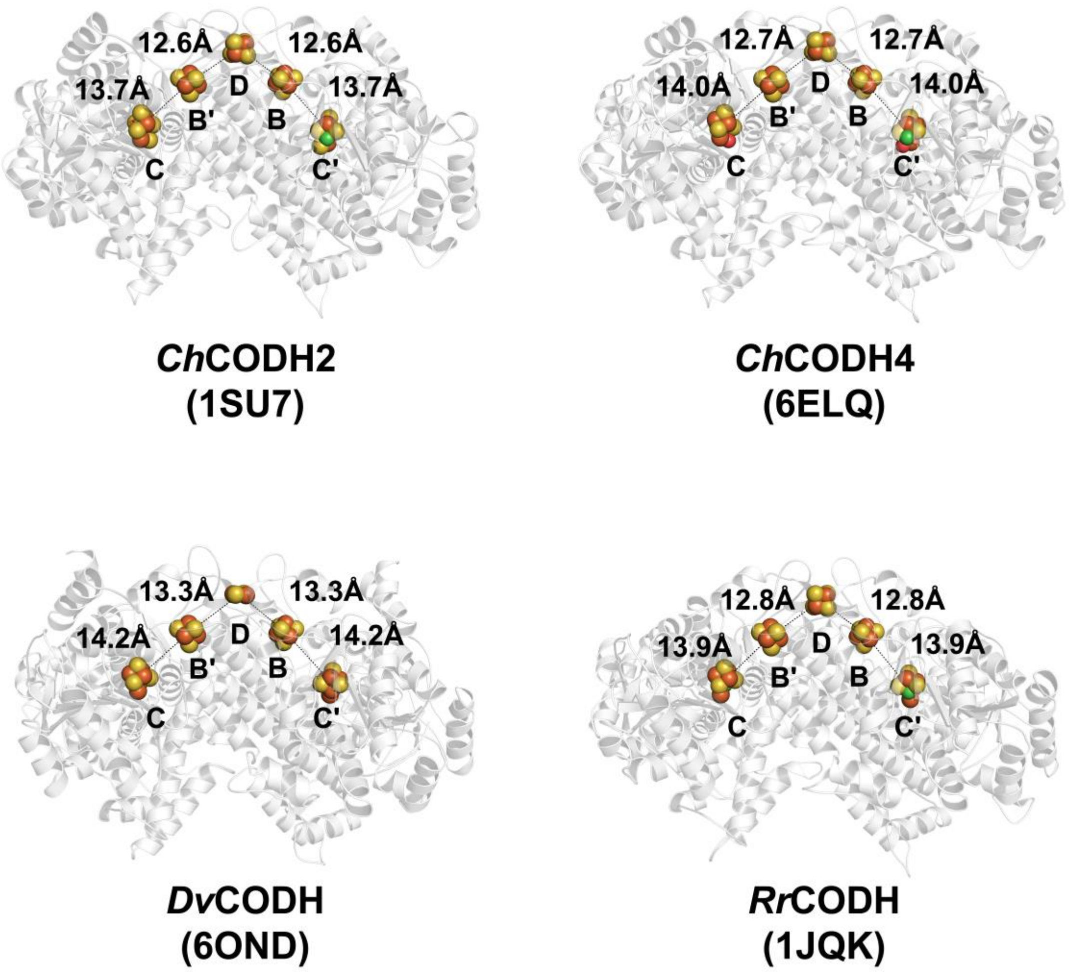

Supplementary Figure 16 | Distance of the putative interacting site for mediators from metal clusters. The distances between each Fe–S cluster (B, C, D) were determined for the four structurally characterized CODHs (*Ch*CODH2[21], *Ch*CODH4[22], *Dv*CODH[25], and *Rr*CODH[23]).

a

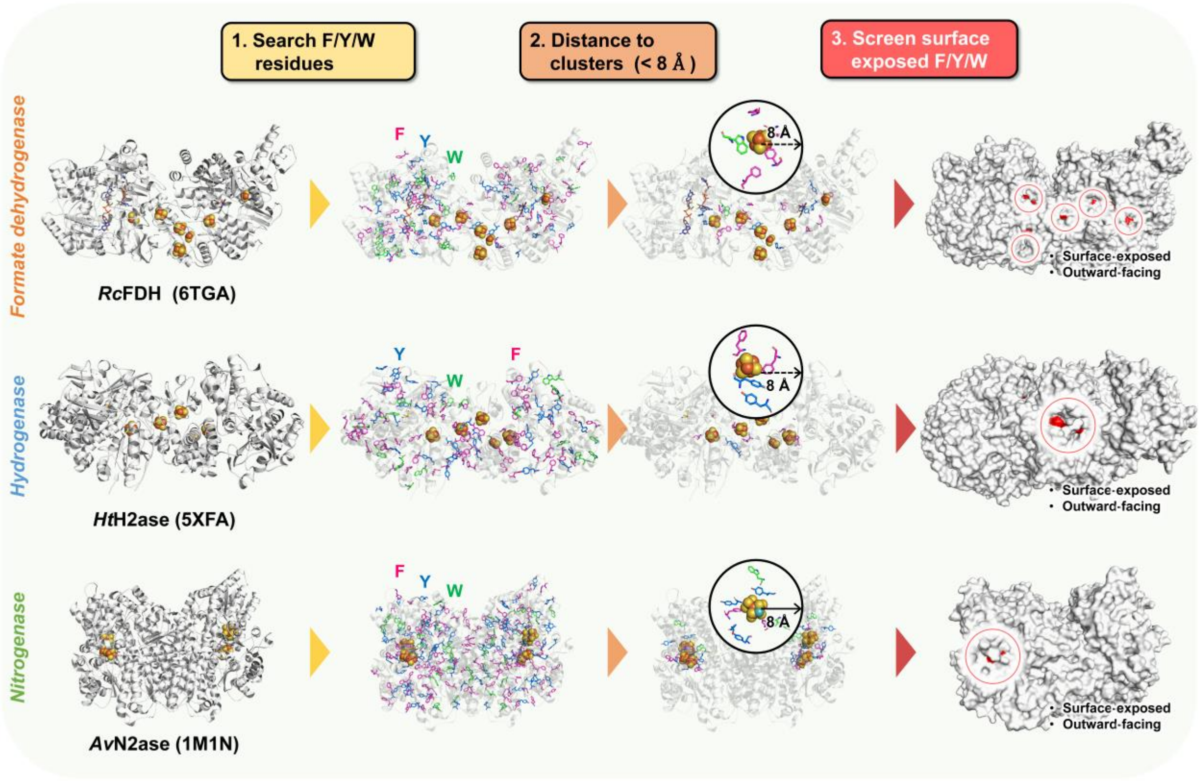

b

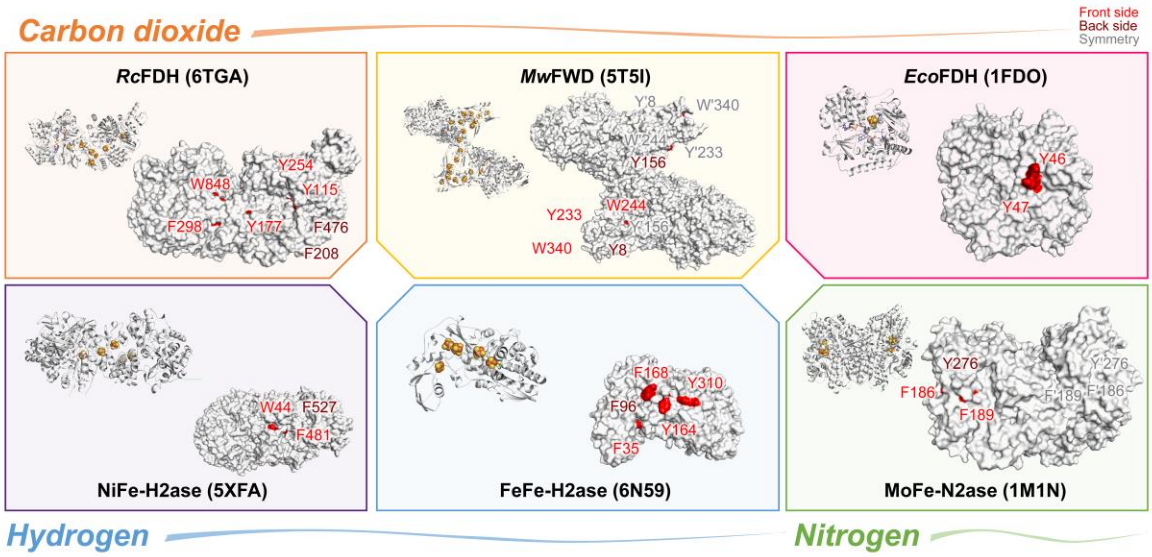

Supplementary Figure 17 | Predicting putative mediator-interaction sites in gas-utilizing enzymes. a, Schematic representation of the workflow for predicting putative mediator-interaction sites. Through a simple method based on specific criteria (surface-exposed F, Y, W residues; 8 Å distance from the cluster; outward-facing side chains), we can specify candidate mediator-interaction sites and their corresponding residues in industrially important metalloenzymes (FDH, H2ase (hydrogenase), N2ase (nitrogenase)). **b,** Predicted residues potentially involved in mediator interactions for gas-utilizing enzymes.

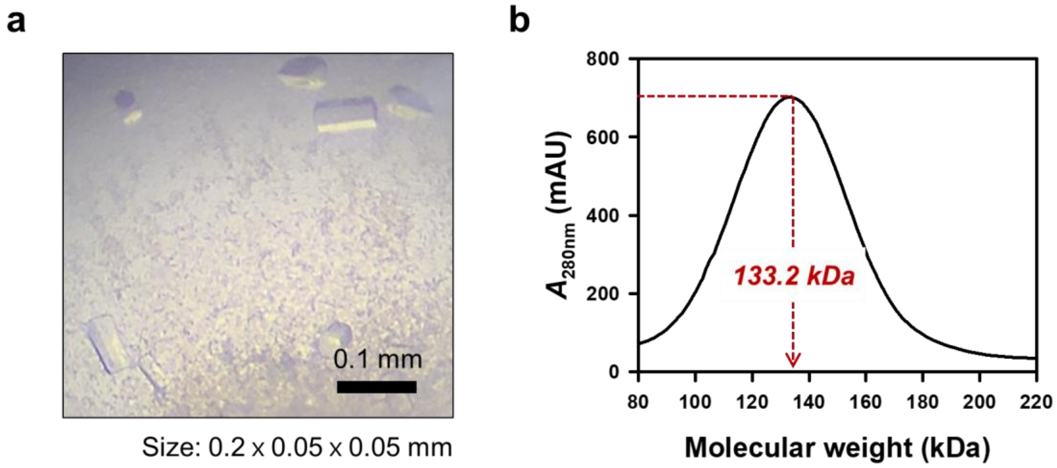

Supplementary Figure 18 | *Ch*CODH2 F41A mutant crystallization. a, Images of F41A mutant crystals with size scale indicated. **b**, Size exclusion chromatography (SEC) analysis of F41A mutant. SEC was used to determine the multimeric state of purified *Ch*CODH2 F41A, with the red dotted line denoting the observed molecular mass (133.3 kDa) compared to the theoretical molecular mass (138 kDa) of the dimeric *Ch*CODH2 WT and F41A.

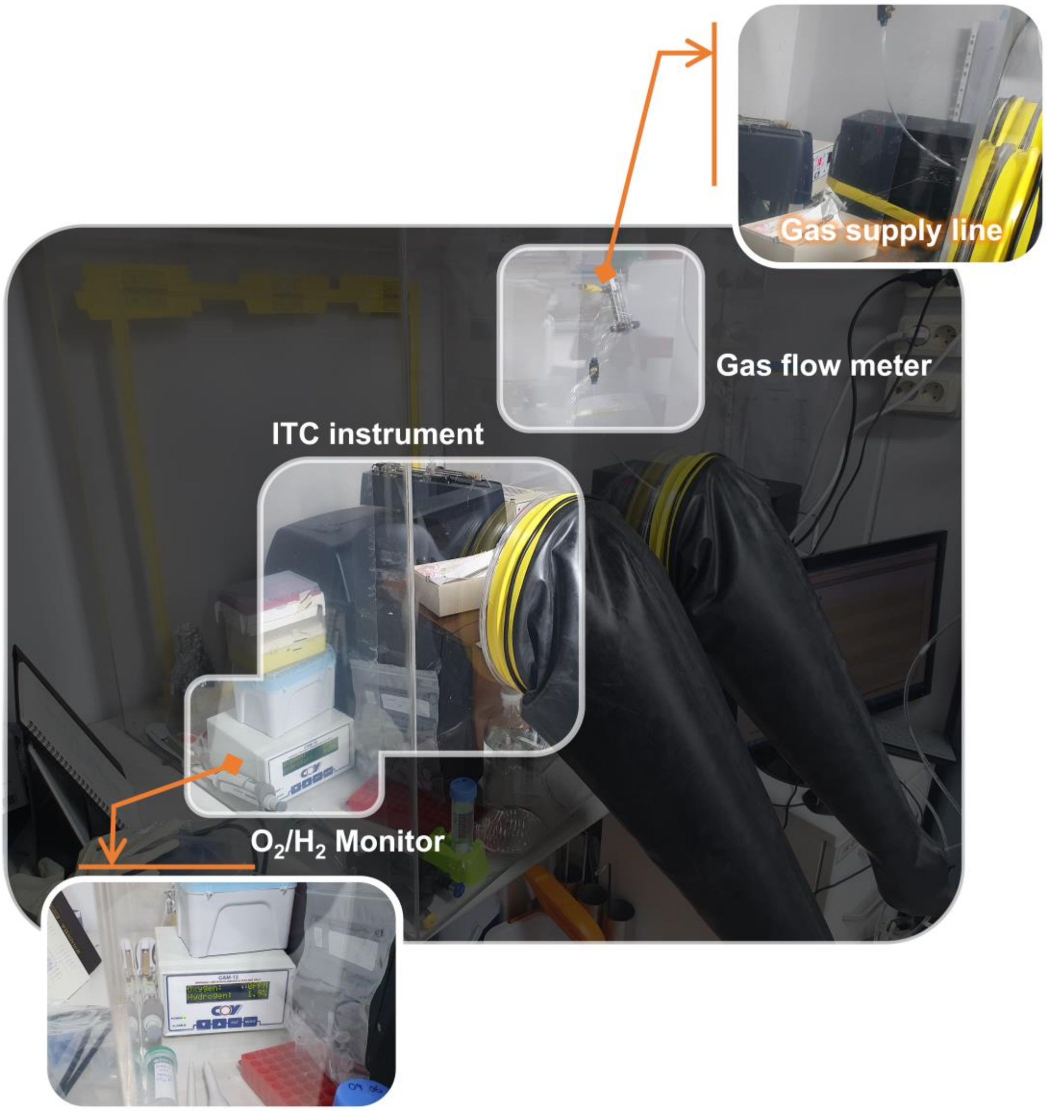

Supplementary Figure 19 | Anaerobic Isothermal Titration Calorimetry (ITC) Analysis. An acryl custom-built glove box was designed and installed with an ITC instrument. An anaerobic environment was established using N_2 gas, and the oxygen level within the box was maintained at a minimal level using a Coy Anaerobic Monitor.

Supplementary References

1. Wittenborn EC, *et al.* Redox-dependent rearrangements of the NiFeS cluster of carbon monoxide dehydrogenase. *eLife* **7**, e39451 (2018).
2. Heo J, Halbleib CM, Ludden PW. Redox-dependent activation of CO dehydrogenase from *Rhodospirillum rubrum*. *Proc. Natl. Acad. Sci. USA* **98**, 7690–7693 (2001).
3. Benvenuti M, *et al.* The two CO-dehydrogenases of *Thermococcus* sp. AM4. *BBA Bioenerg.* **1861**, 148188 (2020).
4. Ruickoldt J, Basak Y, Domnik L, Jeoung JH, Dobbek H. On the kinetics of CO_2 reduction by Ni, Fe-CO dehydrogenases. *ACS Catal.* **12**, 13131–13142 (2022).
5. Lemaire ON, Wagner T. Gas channel rerouting in a primordial enzyme: structural insights of the carbon-monoxide dehydrogenase/acetyl-CoA synthase complex from the acetogen *Clostridium autoethanogenum*. *BBA Bioenerg.* **1862**, 148330 (2021).
6. Grahame DA, Stadtman TC. Carbon monoxide dehydrogenase from *Methanosarcina barker*i: disaggregation, purification, and physicochemical properties of the enzyme. *J. Biol. Chem.* **262**, 3706–3712 (1987).
7. Shin W, Lee S, Shin J, Lee S, Kim Y. Highly selective electrocatalytic conversion of CO_2 to CO at -0.57V (NHE) by carbon monoxide dehydrogenase from *Moorella thermoacetica*. *J. Am. Chem. Soc.* **125**, 14688–14689 (2003).
8. Olson BJSC, Skavdahl M, Ramberg H, Osterman JC, Markwell J. Formate dehydrogenase in *Arabidopsis thaliana*: characterization and possible targeting to the chloroplast. *Plant Sci.* **159**, 205–212 (2000).
9. Slusarczyk H, Felber S, Kula MR, Pohl M. Stabilization of NAD-dependent formate dehydrogenase from *Candida boidinii* by site-directed mutagenesis of cysteine residues. *Eur. J. Biochem.* **267**, 1280–1289 (2000).
10. Cakar MM, *et al.* Engineered formate dehydrogenase from *Chaetomium thermophilum*, a promising enzymatic solution for biotechnical CO_2 fixation. *Biotechnol. Lett.* **42**, 2251–2262 (2020).
11. Fogal S, Beneventi E, Cendron L, Bergantino E. Structural basis for double cofactor specificity in a new formate dehydrogenase from the acidobacterium *Granulicella mallensis* MP5ACTX8. *Appl. Microbiol. Biotechnol.* **99**, 9541–9554 (2015).
12. Shabalin IG, *et al.* Structures of the apo and holo forms of formate dehydrogenase from the bacterium *Moraxella* sp. C-1: towards understanding the mechanism of the closure of the interdomain cleft. *Acta Crystallogr. D Biol. Crystallogr.* **65**, 1315–1325 (2009).

13. Guo X*, et al.* Non-natural cofactor and formate-driven reductive carboxylation of pyruvate. *Angew. Chem. Int. Ed.* **59**, 3143–3146 (2020).
14. Walker LM, Li B, Niks D, Hille R, Elliott SJ. Deconvolution of reduction potentials of formate dehydrogenase from *Cupriavidus necator*. *J. Biol. Inorg. Chem.* **24**, 889–898 (2019).
15. Hartmann T*, et al.* The molybdenum active site of formate dehydrogenase is capable of catalyzing C–H bond cleavage and oxygen atom transfer reactions. *Biochemistry* **55**, 2381–2389 (2016).
16. Axley MJ, Grahame DA. Kinetics for formate dehydrogenase of *Escherichia coli* formate-hydrogenlyase. *J. Biol. Chem.* **266**, 13731–13736 (1991).
17. de Bok FA, Luijten ML, Stams AJ. Biochemical evidence for formate transfer in syntrophic propionate-oxidizing cocultures of *Syntrophobacter fumaroxidans* and *Methanospirillum hungatei*. *Appl. Environ. Microbiol.* **68**, 4247–4752 (2002).
18. Riedererhenderson MA, Peck HD. Properties of formate dehydrogenase from *Desulfovibrio gigas*. *Can. J. Microbiol.* **32**, 430–435 (1986).
19. da Silva SM, Pimentel C, Valente FM, Rodrigues-Pousada C, Pereira IA. Tungsten and molybdenum regulation of formate dehydrogenase expression in *Desulfovibrio vulgaris* Hildenborough. *J. Bacteriol.* **193**, 2909–2916 (2011).
20. Sebban C, Blanchard L, Bruschi M, Guerlesquin F. Purification and characterization of the formate dehydrogenase from *Desulfovibrio vulgaris* Hildenborough. *FEMS Microbiol. Lett.* **133**, 143–149 (1995).
21. Dobbek H, Svetlitchnyi V, Liss J, Meyer O. Carbon monoxide induced decomposition of the active site [Ni-4Fe-5S] cluster of CO dehydrogenase. *J. Am. Chem. Soc.* **126**, 5382–5387 (2004).
22. Domnik L*, et al.* CODH-IV: a high-efficiency CO-scavenging CO dehydrogenase with resistance to O_2. *Angew. Chem. Int. Ed.* **56**, 15466–15469 (2017).
23. Drennan CL, Heo J, Sintchak MD, Schreiter E, Ludden PW. Life on carbon monoxide: X-ray structure of *Rhodospirillum rubrum* Ni-Fe-S carbon monoxide dehydrogenase. *Proc. Natl. Acad. Sci. USA* **98**, 11973–11978 (2001).
24. Doukov TI, Iverson TM, Seravalli J, Ragsdale SW, Drennan CL. A Ni-Fe-Cu center in a bifunctional carbon monoxide dehydrogenase/acetyl-CoA synthase. *Science* **298**, 567–572 (2002).
25. Wittenborn EC*, et al.* Structural insight into metallocofactor maturation in carbon monoxide dehydrogenase. *J. Biol. Chem.* **294**, 13017–13026 (2019).

26. Kim SM*, et al.* O_2-tolerant CO dehydrogenase via tunnel redesign for the removal of CO from industrial flue gas. *Nat. Catal.* **5**, 807–817 (2022).
27. Kim MS*, et al.* CO-dependent H_2 production by genetically engineered *Thermococcus onnurineus* NA1. *Appl. Environ. Microbiol.* **79**, 2048–2053 (2013).
28. Jang J, Jeon BW, Kim YH. Bioelectrochemical conversion of CO_2 to value added product formate using engineered *Methylobacterium extorquens*. *Sci. Rep.* **8**, 7211 (2018).
29. Choi EG, Yeon YJ, Min K, Kim YH. Communication-CO_2 reduction to formate: an electro-enzymatic approach using a formate dehydrogenase from *Rhodobacter capsulatus*. *J. Electrochem. Soc.* **165**, H446–H448 (2018).
30. Seravalli J, Ragsdale SW. ^{13}C NMR characterization of an exchange reaction between CO and CO_2 catalyzed by carbon monoxide dehydrogenase. *Biochemistry* **47**, 6770–6781 (2008).
31. Ragsdale SW, Clark JE, Ljungdahl LG, Lundie LL, Drake HL. Properties of purified carbon monoxide dehydrogenase from *Clostridium thermoaceticum*, a nickel, iron-sulfur protein. *J. Biol. Chem.* **258**, 2364–2369 (1983).
32. Tokmurzin D*, et al.* High temperature flash pyrolysis characteristics of waste plastics (SRF) in a bubbling fluidized bed: effect of temperature and pelletizing. *Fuel* **326**, 125022 (2022).
33. Tokmurzin D*, et al.* Three-dimensional CFD simulation of waste plastic (SRF) gasification in a bubbling fluidized bed with detailed kinetic chemical model. *Energy Convers. Manag.* **267**, 115925 (2022).
34. Chovancova E*, et al.* CAVER 3.0: a tool for the analysis of transport pathways in dynamic protein structures. *PLoS Comput. Biol.* **8**, e1002708 (2012).
35. Jeoung JH, Dobbek H. Carbon dioxide activation at the Ni,Fe-cluster of anaerobic carbon monoxide dehydrogenase. *Science* **318**, 1461–1464 (2007).
36. Feng J, Lindahl PA. Effect of sodium sulfide on Ni-containing carbon monoxide dehydrogenases. *J. Am. Chem. Soc.* **126**, 9094–9100 (2004).
37. Crooks GE, Hon G, Chandonia JM, Brenner SE. WebLogo: a sequence logo generator. *Genome Res.* **14**, 1188–1190 (2004).
