# Supplementary material for: Identifying a key spot for electron mediator-interaction to tailor CO dehydrogenase’s affinity
Source: Nat Commun. 2024 Mar 28;15:2732. doi: 10.1038/s41467-024-46909-1 (PMC10979024; doi:10.1038/s41467-024-46909-1)
Supplement: Supplementary file 3 — Reporting Summary [file 41467_2024_46909_MOESM3_ESM.pdf]

nature portfolio

Corresponding author(s): Yong Hwan Kim

Last updated by author(s): Feb 24, 2024

Reporting Summary

Nature Portfolio wishes to improve the reproducibility of the work that we publish. This form provides structure for consistency and transparency in reporting. For further information on Nature Portfolio policies, see our Editorial Policies and the Editorial Policy Checklist.

Statistics

For all statistical analyses, confirm that the following items are present in the figure legend, table legend, main text, or Methods section.

n/a	Confirmed	
☐	☒	The exact sample size (n) for each experimental group/condition, given as a discrete number and unit of measurement
☐	☒	A statement on whether measurements were taken from distinct samples or whether the same sample was measured repeatedly
☒	☐	The statistical test(s) used AND whether they are one- or two-sided<br>*Only common tests should be described solely by name; describe more complex techniques in the Methods section.*
☐	☒	A description of all covariates tested
☐	☒	A description of any assumptions or corrections, such as tests of normality and adjustment for multiple comparisons
☐	☒	A full description of the statistical parameters including central tendency (e.g. means) or other basic estimates (e.g. regression coefficient) AND variation (e.g. standard deviation) or associated estimates of uncertainty (e.g. confidence intervals)
☒	☐	For null hypothesis testing, the test statistic (e.g. F, t, r) with confidence intervals, effect sizes, degrees of freedom and P value noted<br>*Give P values as exact values whenever suitable.*
☒	☐	For Bayesian analysis, information on the choice of priors and Markov chain Monte Carlo settings
☒	☐	For hierarchical and complex designs, identification of the appropriate level for tests and full reporting of outcomes
☒	☐	Estimates of effect sizes (e.g. Cohen's d, Pearson's r), indicating how they were calculated

Our web collection on statistics for biologists contains articles on many of the points above.

Software and code

Policy information about availability of computer code

Data collection	Data were collected using the Agilent Technologies Cary WinUV Kinetics Application V.5.0.0.999 and the Agilent Chemstation software Version B.04.03. Crystal Data were collected using the Macromolecular Crystallography Data Collector software (MxDC).
Data analysis	Data were analyzed using SigmaPlot Version 10.0 (10.0.0.54), Agilent Technologies Cary WinUV Kinetics Application V.5.0.0.999, Agilent Chemstation software Version B.04.03, PyMOL Molecular Graphics System Version 2.4.0, CAVER PyMOL 3.0 Plugin, GraphPad Prism 7, MEGA Version 10.1.8, HKL2000 v721, CCP4i 7.1.018, PHENIX 1.19.2_4158, and Coot 0.9.8.

For manuscripts utilizing custom algorithms or software that are central to the research but not yet described in published literature, software must be made available to editors and reviewers. We strongly encourage code deposition in a community repository (e.g. GitHub). See the Nature Portfolio guidelines for submitting code & software for further information.

Data

Policy information about availability of data

All manuscripts must include a data availability statement. This statement should provide the following information, where applicable:

- Accession codes, unique identifiers, or web links for publicly available datasets
- A description of any restrictions on data availability
- For clinical datasets or third party data, please ensure that the statement adheres to our policy

Structural data that support the findings of this study have been deposited in the Protein Data Bank (PDB at www.rcsb.org) with the accession codes 8X9D (R57G/N59L apo), 8X9E (R57G/N59L apo in 5% PEG), 8X9F (R57G/N59L with EV), 8X9D (R57G/N59L with BV), and 8X9H (F41C).

Research involving human participants, their data, or biological material

Policy information about studies with human participants or human data. See also policy information about sex, gender (identity/presentation), and sexual orientation and race, ethnicity and racism.

Reporting on sex and gender	N/A
Reporting on race, ethnicity, or other socially relevant groupings	N/A
Population characteristics	N/A
Recruitment	N/A
Ethics oversight	N/A

Note that full information on the approval of the study protocol must also be provided in the manuscript.

Field-specific reporting

Please select the one below that is the best fit for your research. If you are not sure, read the appropriate sections before making your selection.

☒ Life sciences ☐ Behavioural & social sciences ☐ Ecological, evolutionary & environmental sciences

For a reference copy of the document with all sections, see nature.com/documents/nr-reporting-summary-flat.pdf

Life sciences study design

All studies must disclose on these points even when the disclosure is negative.

Sample size	No statistical methods were used to predetermine the sample size. At least three independent experiments were performed in this work. Sample sizes were determined on the basis of previous experiments by considering biological and technical variabilities.
Data exclusions	No data were excluded in this work.
Replication	At least three independent experiments were performed to verify our results. All of the attempts were successful.
Randomization	Randomization was not applicable for the experiments performed, since no statistical comparisons were performed.
Blinding	Blinding was not relevant to our study, since no statistical comparisons were performed.

Reporting for specific materials, systems and methods

We require information from authors about some types of materials, experimental systems and methods used in many studies. Here, indicate whether each material, system or method listed is relevant to your study. If you are not sure if a list item applies to your research, read the appropriate section before selecting a response.

Materials & experimental systems

n/a	Involved in the study
☒	☐ Antibodies
☒	☐ Eukaryotic cell lines
☒	☐ Palaeontology and archaeology
☒	☐ Animals and other organisms
☒	☐ Clinical data
☒	☐ Dual use research of concern
☒	☐ Plants

Methods

n/a	Involved in the study
☒	☐ ChIP-seq
☒	☐ Flow cytometry
☒	☐ MRI-based neuroimaging

Plants

Seed stocks	N/A
Novel plant genotypes	N/A
Authentication	N/A
